# Synthesis of the Bulky Phosphanide [P(Si^*i*^Pr_3_)_2_]^−^ and Its Stabilization of Low-Coordinate Group 12 Complexes

**DOI:** 10.1021/acs.inorgchem.4c03134

**Published:** 2024-10-10

**Authors:** Olivia P. Churchill, Antonia Dase, Laurence J. Taylor, Stephen P. Argent, Nathan T. Coles, Gavin S. Walker, Deborah L. Kays

**Affiliations:** † School of Chemistry, University of Nottingham, University Park, Nottingham NG7 2RD, U.K.; ‡ School of Chemistry, Cardiff University, Main Building, Park Place, Cardiff CF10 3AT, U.K.; § Advanced Materials Research Group, Faculty of Engineering, University of Nottingham, Nottingham NG7 2GA, U.K.

## Abstract

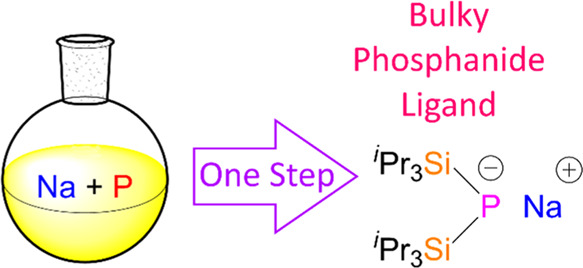

Here, we report an improved synthesis of the bulky phosphanide anion [P(Si^*i*^Pr_3_)_2_]^−^ in synthetically useful yields and its complexation to group 12 metals. The ligand is obtained as the sodium salt NaP(Si^*i*^Pr_3_)_2_**1** in a 42% isolated yield and a single step from red phosphorus and sodium. This is a significantly higher-yielding and safer preparation compared to the previously reported synthesis of this ligand, and we have thus applied **1** to the synthesis of the two-coordinate complexes M[P(Si^*i*^Pr_3_)_2_]_2_ (M = Zn, Cd, Hg). These group 12 complexes are all monomeric and with nonlinear P–M–P angles in the solid state, with DFT calculations suggesting that this bending is due to the steric demands of the ligand. Multinuclear NMR spectroscopy revealed complex second-order splitting patterns due to strong PP’ coupling. This work demonstrates that the synthesis of **1** is viable and provides a springboard for the synthesis of low-coordinate complexes featuring this unusual bulky ligand.

## Abstract 2

The bulky phosphanide anion [P(Si^*i*^Pr_3_)_2_]^−^ has been synthesized as the sodium salt NaP(Si^*i*^Pr_3_)_2_ in a higher-yielding and safer preparation than previous methods. Application of NaP(Si^*i*^Pr_3_)_2_ to the formation of the two-coordinate complexes M[P(Si^*i*^Pr_3_)_2_]_2_ (M = Zn, Cd, Hg) demonstrates the synthetic utility. These group 12 complexes are monomeric, and the deviation from linearity of the P−M−P unit in the solid state is proposed to result from the steric demands of the ligand.

## Introduction

The use of sterically demanding ligands to enforce low-coordination geometries upon d- and f-block metal centers remains an area of interest for inorganic chemists.^1−7^ Such complexes are typically highly reactive and thus capable of acting as a catalyst or a reagent for small molecule reactivity and activation.^3,8−17^ These complexes can also display single molecule magnet (SMM) behavior.^18−27^ The use of amides as versatile, sterically demanding ligands dates to the 1960s, with the use of the [N(SiMe_3_)_2_]^−^ ligand to isolate first the alkali metal silylamides, then the homoleptic Al[N(SiMe_3_)_2_]_3_ and Sn[N(SiMe_3_)_2_]_2_ complexes, as well as several two-coordinate d-block complexes.^28−34^ Since then, a wide array of bulky silylamide ligands such as [N(Dipp)(SiMe_3_)]^−^, [N(SiHMe_2_)_2_]^−^, and [N(SiPh_2_Me)_2_]^−^ have been developed and utilized in complexation reactions with metals across the periodic table.^4,16,35−44^ More recently, the exceedingly bulky KN(Si^*i*^Pr_3_)_2_ has been applied to the synthesis of linear f-block species, which display large magnetic anisotropy and have the potential for extremely high *U*_eff_ values (*U*_eff_ = barrier to magnetization),^45−47^ as well as group 2 Lewis acidic cations.^48^ Most recently, investigations of (^*t*^Bu_3_Si)_2_NH showed the amine to be resistant to deprotonation even by ^*n*^BuLi/KO^*t*^Bu superbase mixtures. However, the coordination of [N(Si^*t*^Bu_3_)_2_]^−^ to Cs was achieved through the reaction of (*^t^*Bu_3_Si)_2_NH with Cs^0^/THP electride solution (THP = tetrahydropyran). The resulting Cs(NSi^*t*^Bu_3_)_2_ complex was shown to undergo a metathesis reaction when reacted with LiI.^49^

While bulky silylamides are relatively well established, the corresponding phosphorus analogues have received considerably less attention. Previous studies of the [P(SiMe_3_)_2_]^−^ ligands have afforded dimeric or polymeric structures. The substitution reactions between M[N(SiMe_3_)_2_]_2_ (M = Zn, Cd, Hg, Sn, Pb, and Mn) with two equivalents of (Me_3_Si)_2_PH lead to the formation of the dimeric [M(P(SiMe_3_)_2_{μ_2_-P(SiMe_3_)_2_}]_2_ complexes. In the case of the reaction with manganese, (THF)Mn[N(SiMe_3_)_2_] was used and the resulting phosphanide complex contained a three-coordinate and a four-coordinate manganese center bearing one THF ligand.^50^ Dimeric Li(THF)_2_P(SiMe_3_)_2_, tetrameric Li(THF)_0.5_P(SiMe_3_)_2_, and hexameric LiP(SiMe_3_)_2_ complexes were prepared from the reaction of P(SiMe_3_)_3_ with ^*n*^BuLi in THF or cyclopentane,^51,52^ while polymeric, ladder-type structures of the heavier alkali metals with the general formula [(THF)AP(SiMe_3_)_2_]_∞_ (A = K, Rb, Cs) were prepared from the reaction of P(SiMe_3_)_3_ with the corresponding alkali metal *tert*-butoxide (AO^*t*^Bu).^53^ To the best of our knowledge, the only two-coordinate metal bis(silylphosphanido) complexes to date are M[P(SiPh_3_)_2_]_2_ (M = Zn, Cd, Hg), prepared by Matchett et al.^54^ Here, the higher steric demands of the –SiPh_3_ group offset the larger P atom, allowing for the isolation of monomeric species. Thus, we propose that the phosphorus analogue of the aforementioned [N(Si^*i*^Pr_3_)_2_]^−^ ligand is of considerable interest due to its steric bulk, which should allow the isolation of monomeric complexes. While the [P(Si^*i*^Pr_3_)_2_]^−^ ligand is known, it has scarcely been studied due to difficulties in its preparation. Westerhausen et al. prepared the Li salt [(THF)LiP(Si^*i*^Pr_3_)_2_]_2_ by first reacting ^*n*^BuLi with PH_3_ in the presence of DME (DME = 1,2-dimethoxyethane) to obtain (DME)LiPH_2_ in an 82–91% yield.^55,56^ This was then reacted with ^*i*^Pr_3_SiCl to afford ^*i*^Pr_3_SiPH_2_ (64%), with (^*i*^Pr_3_Si)_2_PH obtained as a minor byproduct (13%).^57^ Further reaction of the minor product (^*i*^Pr_3_Si)_2_PH with ^*n*^BuLi in THF afforded [(THF)LiP(Si^*i*^Pr_3_)_2_]_2_ in an 83% yield,^58^ giving an overall yield from PH_3_ of at most 9.8%. Given the difficult and low-yielding synthesis, [(THF)LiP(Si^*i*^Pr_3_)_2_]_2_ was used to prepare only one complex, [(THF)_4_Li][(^*i*^Pr_3_Si)_2_PW(CO)_5_]. As such, the potential of this ligand is largely unexplored.

Herein, we present a much-improved synthesis of an alkali metal complex of this ligand, the Na^+^ salt NaP(Si^*i*^Pr_3_)_2_ (**1**), which was obtained in a 42% isolated yield and in a single step. This has allowed us to prepare the family of group 12 complexes M[P(Si^*i*^Pr_3_)_2_]_2_ (M = Zn (**2**), Cd (**3**), Hg (**4**)), by salt metathesis reactions, demonstrating the synthetic utility of this ligand precursor.

## Results and Discussion

### Synthesis of NaP(Si^*i*^Pr_3_)_2_**1**

To obtain a more direct route to the [P(Si^*i*^Pr_3_)_2_]^−^ anion than previously reported,^58^ we looked to the synthesis of P(Si^*i*^Pr_3_)_3_ published by von Hänisch. Here, red phosphorus was reacted with NaK in refluxing DME to generate (Na/K)_3_P, which was subsequently reacted with ^*i*^Pr_3_SiCl.^59^ Since P(SiMe_3_)_3_ can be converted to (Me_3_Si)_2_PH by hydrolysis or methanolysis,^60,61^ we postulated that it could be possible to obtain (^*i*^Pr_3_Si)_2_PH in a similar manner. However, the use of a highly pyrophoric NaK alloy was a safety concern. To mitigate this, we instead used Na with 10 mol % naphthalene as an electron-transfer agent.^62^ This method has been used previously to generate Na_3_P *in situ*(63,64) for the preparation of tris(trimethylsilyl)phosphine, P(SiMe_3_)_3_.^65^

**Figure 1:**
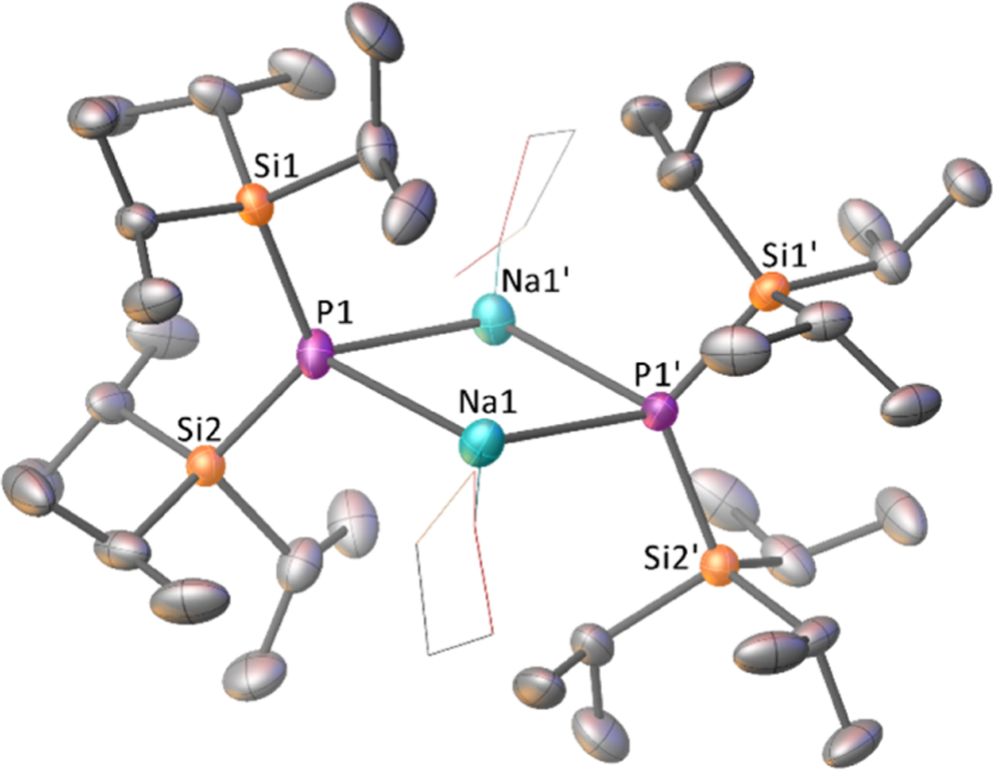
Single-crystal X-ray diffraction structure of [(THF)NaP(Si^i^Pr_3_)_2_]_2_**1a**. Coordinated THF represented as a wireframe, minor disorder components, and hydrogen atoms omitted for clarity. Thermal ellipsoids are set to the 50% probability. Atoms marked with ‘ are obtained using the following symmetry operation: 1–*x*, +*y*, –*z*. Selected bond lengths (Å) and angles (deg): Na1–P1 2.8039(11), Na1–P1’ 2.806(1), Na–O1A 2.200(6), Na–O1B 2.203(6), Na–O1C 2.309(3), Na1•••Na1’ 3.4586(18), P1–Si1 2.2207(8), P1–Si2 2.2163(7), P1–Na1–P1’ 103.84(3), Na1–P1–Na1’ 76.15(3), and Si1–P1–Si2 120.56(3).

In our initial testing, we found that it was necessary to reflux the Na/naphthalene and red phosphorus for 24 h in DME; otherwise, the resulting product contained significant amounts of unreacted ^*i*^Pr_3_SiCl and (^*i*^Pr_3_Si)_2_. This is believed to occur due to the incomplete formation of Na_3_P and the presence of unreacted Na. When monitoring the reaction by ^31^P NMR spectroscopy, we found that a mixture of P-containing species was formed, including P(Si^*i*^Pr_3_)_3_ and (^*i*^Pr_3_Si)_2_PH, which were identified by comparison with the literature.^58^ Another significant ^31^P NMR signal was observed at −378 ppm (compound **1**). By removing the DME *in vacuo*, then extracting the resulting residue in hexane or toluene, it was possible to precipitate **1** from the reaction mixture as a white pyrophoric solid, while P(Si^*i*^Pr_3_)_3_, (^*i*^Pr_3_Si)_2_PH, and other P-containing byproducts remained in solution. ^1^H, ^13^C{^1^H}, ^31^P, and ^31^P{^1^H} NMR spectroscopy of **1** suggested that the complex contained a – P(Si^*i*^Pr_3_)_2_ moiety with minor residual solvent peaks (see Supporting Information Figures S2 and S3). While it was not possible to obtain crystals of **1** suitable for single-crystal X-ray diffraction studies, crystals of [(THF)NaP(Si^*i*^Pr_3_)_2_]_2_ (**1a**) were obtained when a reaction mixture containing **1** was dissolved in C_6_D_6_ and THF (Figure 1) and left at room temperature for 4 weeks. Based on this structure and the NMR spectroscopic data, we propose that **1** corresponds to NaP(Si^*i*^Pr_3_)_2_. Due to the very high sensitivity of **1**, it was not possible to obtain high-resolution mass spectrometric data on this compound.

**Scheme 1:**
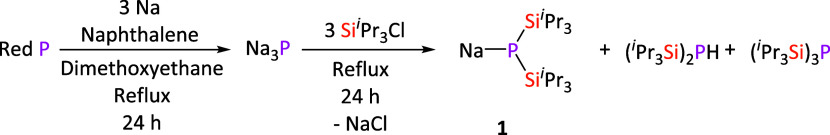
Optimized Synthesis of NaP(Si^i^Pr_3_)_2_ (**1**)

Given that our aim had been to convert P(Si^*i*^Pr_3_)_3_ to the [P(Si^*i*^Pr_3_)_2_]^−^ anion via a multistep process, the observation of **1** was quite exciting. Here, we directly formed a phosphanide anion in a single step and purified it by precipitation and filtration. Thus, we focused on optimizing the synthesis to maximize the yield of **1**, rather than P(Si^*i*^Pr_3_)_3_. This led to the development of the methodology shown in Scheme 1. Note that attempts with a 2:1 stoichiometry of ^*i*^Pr_3_SiCl:Na_3_P resulted in an increase in the quantity of (^*i*^Pr_3_Si)_2_PH produced relative to the desired NaP(Si^*i*^Pr_3_)_2_ (only 13% NaP(Si^*i*^Pr_3_)_2_ observed by ^31^P NMR of the crude reaction mixture); thus, a 3:1 reaction stoichiometry was found to perform best. Na and 10 mol % naphthalene were refluxed in DME for 24 h, after which ^*i*^Pr_3_SiCl was added and the reaction was heated for a further 24 h. After filtration to remove insoluble impurities, the DME was removed *in vacuo*, and the resulting oil was extracted into toluene. This precipitated **1**, which was isolated by filtration in a 42% yield with sufficient purity for further synthesis. The crude reaction mixture showed the formation of **1**, (^*i*^Pr_3_Si)_2_PH, and P(Si^*i*^Pr_3_)_3_ in an approximate 1:0.28:0.08 ratio (see Supporting Information Figure S6). As (^*i*^Pr_3_Si)_2_PH has been previously shown to be readily converted to [(THF)LiP(Si^*i*^Pr_3_)_2_]_2_,^58^ which can also be used in transmetalation reactions, it is suggested that isolation of this byproduct would further increase the yield of usable phosphanide precursors from this reaction. Note that, concurrent with our reported work, the Mills group has developed a similar (albeit lower-yielding) synthesis of **1**.^66^

### Synthesis of Group 12 Complexes **2**–**4**

**Scheme 2:**
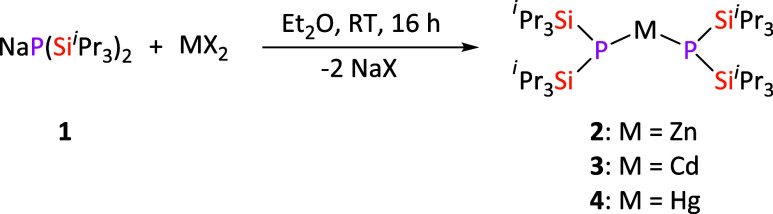
Synthesis of Group 12 Bis(silylphosphanido) Complexes **2**–**4**. MX_2_ = ZnCl_2_, CdI_2_, HgBr_2_

The two-coordinate group 12 complexes **2**–**4** were prepared by the metathesis reaction of **1** with the appropriate metal halide (ZnCl_2_, CdI_2_, and HgBr_2_) in diethyl ether (Scheme 2). The resulting complexes were isolated as white crystalline solids in moderate to good yields (40–57%) after extraction and recrystallization from *n*-hexane. For the formation of **3**, it was necessary to use CdI_2_, as reactions between **1** and CdCl_2_ in diethyl ether led to precipitation of Cd(0). Complexes **2**–**4** are air- and moisture-sensitive and were characterized by single-crystal X-ray diffraction and multinuclear NMR spectroscopy. Complex **2** was also characterized by HRMS and CHN microanalysis; the high toxicity of complexes **3** and **4** precluded their analysis by these methods.

**Figure 2:**
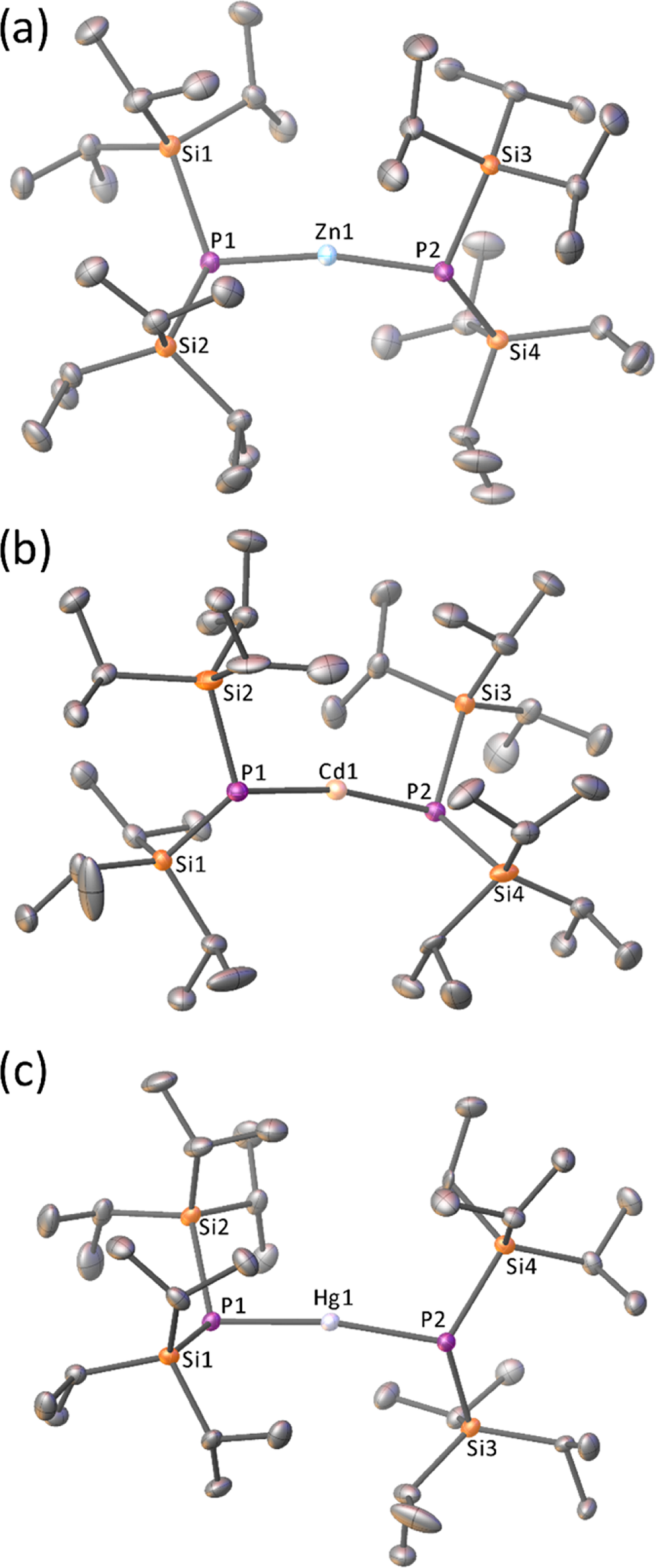
View of the single-crystal X-ray diffraction structures of (a) **2**, (b) **3**, and (c) **4**. Hydrogen atoms and minor disorder components of **3** and **4** have been omitted for clarity. Thermal ellipsoids are shown at a 50% probability.

Crystals of **2**–**4** suitable for single-crystal X-ray diffraction were obtained from the storage of saturated *n*-hexane solutions at −30 °C (Figure 2). A polymorph structure of **2** (**2a**) was also obtained by slow evaporation from diethyl ether (Figure S1). **2** and **2a** crystallize in the same space group (P1̅) but with a different unit cell (Table S1) and with significantly different P1–Zn1–P2 angles [168.747(12)° vs 163.593(18)°]. All structures show **2**–**4** to be monomeric and two-coordinate in the solid state. The M1–P1 and M1–P2 bond lengths (Table 1) are similar to those seen in the terminal silylphosphanido groups in [M(P(SiMe_3_)_2_{μ_2_-P(SiMe_3_)_2_}]_2_ (Zn–P_t_ 2.295(1) Å, Cd–P_t_ 2.459(1) Å, and Hg–P_t_ 2.402(1) Å).^50^ The P–Si bond lengths differ slightly between complexes [2.2479(4)–2.2657(8) Å; Table 1] but are consistent with P–Si single bonds, with little evidence of the P–Si double bond character [typical P=Si distances 2.062(1)–2.158(2) Å].^67^ The sum of the angles around each phosphorus center (∑°; Table 1) is also consistent with an sp^3^-hybridized P atom (*i.e*. no P=Si bond character). It has been postulated that the unusually wide Si–N–Si and Si–O–Si bond angles of the bis(silyl)amides and ethers arise from negative hyperconjugation, with increasing steric bulk of the ligands leading to a further widening of these bonds.^49^ In the case of bis(triisopropylsilyl)phosphanide complexes **1a**–**4**, the Si–P–Si bond angles are more acute [117.09(15)–125.903(18)°] than those observed for the bis(triisopropylsilyl)amide lanthanide complexes Ln[N(Si^*i*^Pr_3_)_2_]_2_ (typical Si–P–Si bond angles 137.6–139.8° for Ln = Sm, Eu, Tm, Yb).^45,46^ An increase in the Si–P–Si bond angles arises when comparing complexes **1a** and **2**–**4** with the hexameric [LiP(SiMe_3_)_2_]_6_ complex [Si–P–Si 107.8(1)–108.6(1)°]^52^ and the terminal silylphosphanide groups of [M(P(SiMe_3_)_2_{μ_2_-P(SiMe_3_)_2_}]_2_ [Si–P_t_–Si 106.2(1)° (Zn); 106.6(1)° (Cd); 107.2(1)° (Hg); 100.3(4)° (Pb); 106.6(1), 105.1(1)° (Mn)].^50^ Cd[P(SiPh_3_)_2_]_2_ prepared by Matchett et al. also displays a small Si–P–Si bond angle [107.7(1)°] attributed to the lack of substantial steric interactions between the SiPh_3_ substituents.^54^ The limited data for bis(silyl)phosphanide complexes hinder the ability to establish a correlation between Si–P–Si bond angles and the steric demands of the ligand. All of the complexes exhibit a nonlinear P–M–P unit, with this angle increasing from Zn > Cd > Hg (Table 1).

**Table 1:** Selected Bond Lengths (Å) and Angles (deg) for M[P(Si^i^Pr_3_)_2_]_2_ (M = Zn (**2**), Cd (**3**), Hg (**4**))^a^

	**2**	**2a**	**3**	**4**
M1–P1	2.2291(3)	2.2309(4)	2.4213(7)	2.3946(5)
M1–P2	2.2234(4)	2.2562(4)	2.4216(7)	2.3930(5)
P1–Si1	2.2532(4)	2.2629(4)	2.2571(7)	2.2614(7)
P1–Si2	2.2537(4)	2.2550(5)	2.2487(7)	2.2657(8)
P2–Si3	2.2456(4)	2.2598(5)	2.2557(8)	2.2644(7)
P2–Si4	2.2479(4)	2.2527(4)	2.2570(6)	2.2541(8)
P1–M1–P2	168.747(12)	163.593(18)	169.215(19)	170.086(16)
Si1–P1–Si2	125.9(1)	120.9(1)	120.5(1)	119.5(2)*
				123.9(2)*
Si3–P2–Si4	123.0(1)	120.9(1)	121.6(1)	117.1(2)*
				124.1(2)*
∑° around P1	334.14(2)	337.05(13)	330.52(3)	325.73(3)
∑° around P2	339.76(2)	322.26(13)	324.87(3)	331.16(3)

a Complexes **2**, **3**, and **4** were crystallized from *n*-hexane. **2a** is a polymorph of **2** crystallized from diethyl ether. Values marked with an asterisk correspond to disorder-modeled components.

### Computational Investigations

Given that closed-shell, two-coordinate metal complexes are frequently linear,^68−76^ although nonlinear species are known,^74,77−80^ and that the only previous group 12 bis(silylphosphanido) complex to be structurally characterized (Cd[P(SiPh_3_)_2_]_2_) was linear,^54^ DFT calculations were used to probe the reasons for the deviation from linearity for **2**–**4** in the solid state. Geometry optimizations were performed on **2**–**4** (PBE0/SARC-ZORA-TZVP for Cd and Hg, PBE0/ZORA-def2-TZVP for all other atoms).^81−88^ Grimme’s D3 dispersion corrections were applied to all optimizations.^89,90^ The optimized structures were in good agreement with those determined experimentally and in all cases reproduced the nonlinear P–M–P (M = Zn, Cd, Hg) bond angles (Table S2). Models of **2**–**4** were also optimized with a 180° P–M–P bond angle restraint, affording linear models (**2′**, **3′**, **4′**). These linear models were found to be significantly *less* thermodynamically stable than the bent structures, with linearization energies (Δ*E*_lin_) of 13.3 kcal mol^–1^ for Zn, 10.7 kcal mol^–1^ for Cd, and 8.9 kcal mol^–1^ for Hg.^77^ The linear structures show a significant distortion about the P atoms, with asymmetry in the M–P–Si angles (Figure S31 and Table S2). By contrast, the M–P–Si groups in the nonlinear optimized structures were more symmetric (Figure S32 and Table S2). This suggests that the bent P–M–P bond angles are a consequence of the steric demands of the [P(Si^*i*^Pr_3_)_2_]^−^ ligands. To fit these ligands around the metal, it is necessary to distort at *either* the metal center *or* the P atoms, with the distortion at the metal being more favorable. The solid-state structure of Cd[P(SiPh_3_)_2_]_2_ shows relatively symmetric Cd–P–Si angles (100.9(2)°, 98.2(1)°) and a linear P–Cd–P angle,^54^ suggesting that a smaller ligand removes the need for distortion. Geometry optimization (without restraints) of the less sterically demanding Cd[P(SiMe_3_)_2_]_2_, starting from linear and nonlinear geometries, afforded both linear (P–Cd–P = 179.9°) and near-linear (P–Cd–P = 177.5°) molecules. These two geometries showed near-identical energies (Δ*G* = 0.1 kcal mol^–1^), suggesting that there is little energetic difference between these two coordination environments for the less sterically demanding [P(SiMe_3_)_2_]^−^ ligand.

### NMR Spectroscopic Analysis

**Figure 3:**
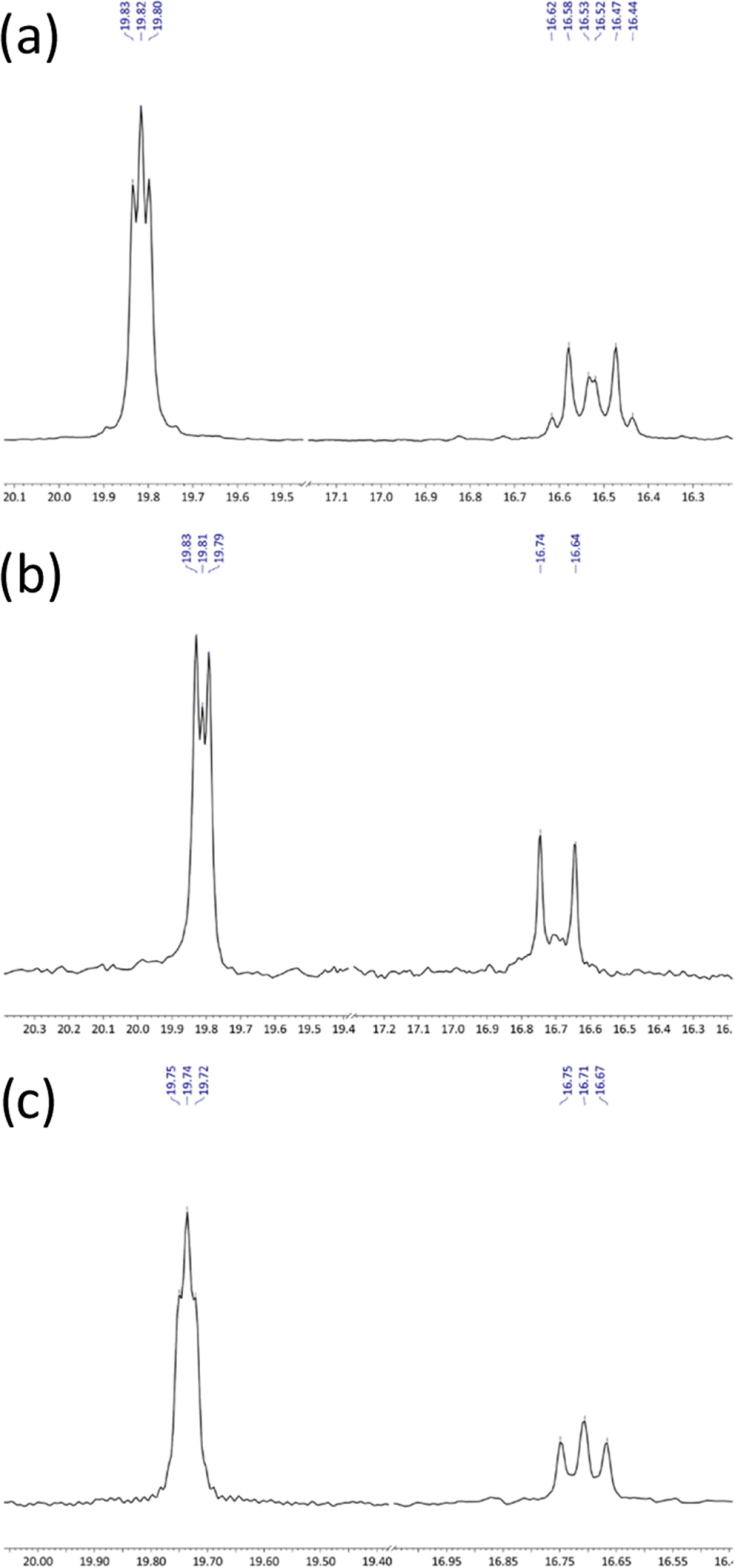
^13^C{^1^H} NMR spectra of (a) **2**, (b) **3**, and (c) **4**, showing the extent of virtual coupling.

**Figure 4:**
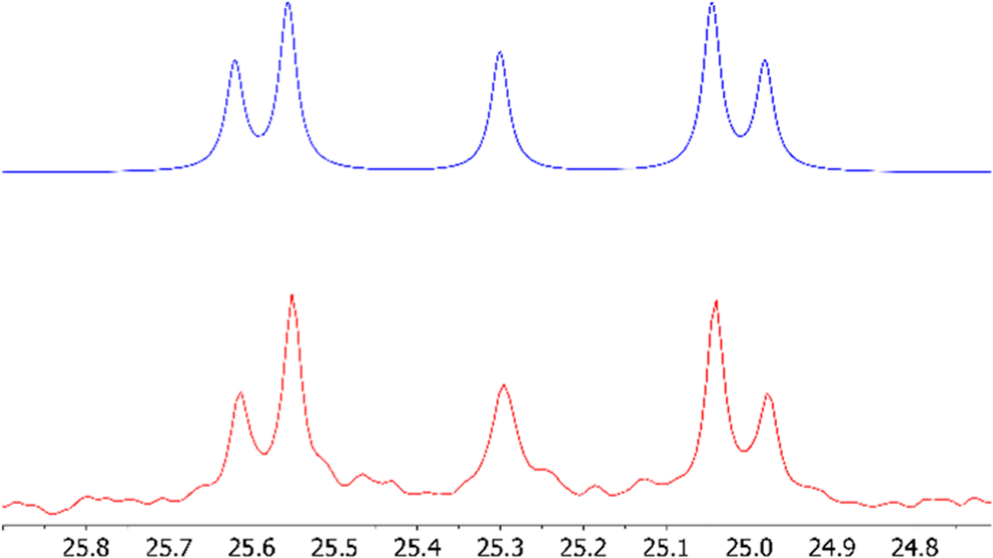
Experimental (red) and simulated (blue) ^29^Si{^1^H} NMR spectra of **4**, modeled using the parameters ^1^*J*_SiP_ = 50.6 Hz, ^3^*J*_SiP’_ = 0.0 Hz, and ^2^*J*_PP’_ = 19.0 Hz.

The ^13^C{^1^H} NMR spectra of **2**–**4** (Figure 3) and the ^29^Si{^1^H} NMR spectrum of **4** (Figure 4) show evidence of second-order effects due to strong virtual coupling between the ^31^P nuclei (^2^*J*_PP’_). Similar effects have been reported in the literature for analogous phosphorus–carbon ABX and AA’X systems.^91−93^ Despite the different appearances of the ^13^C{^1^H} NMR signals, the ^2^*J*_CP_ and ^3^*J*_CP_ coupling constants are similar across the series [^2^*J*_CP_ = 10.7 Hz (**2**), 10.3 Hz (**3**), 10.2 Hz (**4**); ^3^*J*_CP_ = 3.6 Hz (**1**) 3.7 Hz (**2**), 3.5 Hz (**4**)]. This indicates that the differences between **2** and **4** are likely caused by the changing magnitude of ^2^*J*_PP’_ across the series.

While the ^29^Si{^1^H} NMR spectra of **2** and **3** show apparent doublets, that of **4** is more complex, consistent with an AA’X spin system with virtual coupling. This spectrum was well simulated with parameters of ^1^*J*_SiP_ = 50.6 Hz, ^3^*J*_SiP′_ = 0.0 Hz, and ^2^*J*_PP′_ = 19.0 Hz (Figure 4). This ^2^*J*_PP′_ coupling of 19.0 Hz was also used to successfully simulate the ^13^C{^1^H} NMR signals of **4** (see Supporting Information Figures S25 and S26), further supporting this value for ^2^*J*_PP′_.

Also of note are the ^29^Si satellites in the ^31^P{^1^H} NMR spectra of **2**–**4**. While **2** and **4** show apparent ^29^Si satellites, the measured coupling from these satellite peaks does not match that found in the ^29^Si{^1^H} NMR spectra. This is likely due to the presence of one spin-active ^29^Si nucleus causing the two ^31^P nuclei to become magnetically inequivalent such that the satellite signal is not a simple doublet. For **3**, the measured ^2^*J*_SiP_ from the satellites does match the ^29^Si{^1^H} NMR spectrum, suggesting that the two P atoms are (closer to) magnetically equivalent in **3**. The ^31^P{^1^H} NMR signal for **4** (δ_P_ = −209 ppm) occurs significantly downfield of the signals for **2** or **3** (δ_P_ = −288 and −284 ppm, respectively), which is consistent with previously published group 12 bis(silylphosphanide) complexes.^54^ The ^113^Cd and ^199^Hg NMR spectra of **3** and **4** both appear as triplets, with large couplings to phosphorus (^1^*J*_CdP_ = 350 Hz, ^1^*J*_HgP_ = 408 Hz).

## Conclusions

We present the first one-step synthesis of a source of the phosphanide anion [P(Si^*i*^Pr_3_)_2_]^−^, in the form of NaP(Si^*i*^Pr_3_)_2_ (**1**). Complex **1** was obtained in a 42% isolated yield, far higher than that of the previously reported Li phosphanide [(THF)LiP(Si^*i*^Pr_3_)_2_]_2_, thereby offering a significantly improved route to this ligand for synthetic investigations. [(THF)NaP(Si^*i*^Pr_3_)_2_] (**1a**), obtained from the solvation of **1** in THF, was characterized by single-crystal X-ray diffraction. With this synthetically useful methodology for **1**, we were able to complex this sterically demanding phosphanide ligand to Zn, Cd, and Hg, affording the novel two-coordinate complexes **2**–**4**. Single-crystal X-ray diffraction revealed that complexes **2**–**4** all show significant deviations from linearity in the solid state, with DFT calculations suggesting that this is due to the steric demands of the ligand. ^13^C{^1^H} and ^29^Si{^1^H} NMR spectroscopy of these ligands revealed strong second-order effects, suggesting the presence of virtual coupling between the two ^31^P nuclei in these complexes. These studies show that [P(Si^*i*^Pr_3_)_2_]^−^ is now an accessible bulky, monodentate, monoanionic ligand.

## Experimental Section

### General Materials and Methods

All products described were synthesized with the rigorous exclusion of air and water using standard air-sensitive-handling techniques, which included benchtop operations (Schlenk line) and glovebox techniques, under argon and nitrogen, respectively. Solvents (*iso*-hexane, diethyl ether, THF, toluene) were collected from the in-house dry-solvent towers, degassed, and stored over 3 Å molecular sieves. *n*-Hexane was purchased extra-dry over molecular sieves from Thermo Scientific Chemicals, degassed, and stored over a second batch of 3 Å molecular sieves. DME was dried over CaH_2_, distilled, degassed, and stored over 3 Å molecular sieves. C_6_D_6_ was dried over potassium, deuterated pyridine-d_5_ was dried over CaH_2_, and THF-*d*_8_ was dried over sodium; all of these were distilled, degassed, and stored over 3 Å molecular sieves in the glovebox prior to use.

Anaerobic samples for NMR spectroscopy were prepared using glovebox techniques and sealed in J. Young′s tap-modified borosilicate glass NMR tubes. NMR data were collected on either a Bruker AV400, AV(III)400, or AV(III) 500 spectrometer. Chemical shifts are quoted in ppm relative to TMS (^1^H, ^13^C{^1^H}, ^29^Si{^1^H}) or H_3_PO_4_ (85% in D_2_O, ^31^P, and ^31^P{^1^H}). ^113^Cd and ^199^Hg NMR chemical shifts are quoted in ppm relative to CdMe_2_ and HgMe_2_, respectively, using 0.1 M Cd(ClO_4_)_2_/D_2_O and 1.0 M Hg(ClO_4_)_2_/D_2_O solutions as external calibrants.

Anaerobic mass spectrometry samples for **2** were prepared under an argon atmosphere by flame-sealing the sample inside glass capillaries. Each sample was then opened and introduced immediately to a Bruker Impact II spectrometer with an APCI II source and a Direct Insertion Probe.

ATR-IR of solid samples were collected using a Bruker α FTIR spectrometer, using a resolution of 2 cm^–1^, a frequency range of 500–4000 cm^–1^, and a spectral average of 32 scans. These spectra were collected inside a nitrogen-filled glovebox. A background of the atmosphere was obtained prior to each data collection.

Elemental microanalyses were performed on an Exeter Analytical CE-440 Elemental Analyzer with samples combusted at 975 °C prior to measurement.

**Caution:** cadmium and mercury compounds are highly toxic, and great care must be taken in their manipulation.

#### Synthesis of NaP(Si^*i*^Pr_3_)_2_ (**1**)

Na (1.50 g, 65.3 mmol) and red phosphorus (0.63 g, 20.3 mmol) were suspended in DME (150 mL), and naphthalene (100 mg, 0.8 mmol) was added. The solution was refluxed for 24 h. A solution of ^*i*^Pr_3_SiCl (12.2 g, 65.3 mmol) in DME (50 mL) was added dropwise to the Na_3_P solution at room temperature. The resulting suspension was refluxed for a further 24 h before allowing to cool to room temperature. The suspension was filtered to remove insoluble impurities, and the solvent was removed *in vacuo*. The resulting residue was extracted with toluene (200 mL), causing the precipitation of **1** as a pyrophoric white solid, which was isolated by filtration (3.12 g, 8.5 mmol, 42%). It should be noted that samples of **1** contained trace impurities of secondary phosphine (^*i*^Pr_3_Si)_2_PH. ^1^H NMR (pyridine-d_5_, 400 MHz): δ 1.49–1.44 (m, 42H, ^*i*^Pr (C*H*_3_)_2_ and ^*i*^Pr (C*H*)). ^13^C{^1^H} NMR (pyridine-d_5_, 101 MHz): δ 22.5 (d, ^3^*J*_CP_ = 3.7 Hz, *C*H_3_), 18.4 (d, ^2^*J*_CP_ = 9.9 Hz, *C*H). ^31^P NMR (pyridine-d_5_, 162 MHz): δ – 378.3 (s). ^31^P{^1^H} NMR (pyridine-d_5_, 162 MHz): δ – 378.3 (s). ATR-FTIR ν_max_ (cm^–1^) 2936 (br, CH), 2857 (br, CH), 1460 (s, CH), 1358 (w), 1070 (m), 1012 (s), 1008 (w), 990 (w), 876 (s), 650 (s), 621 (s), 556 (s), 513 (s).

#### Synthesis of (THF)NaP(Si^*i*^Pr_3_)_2_ (**1a**)

Single crystals of the THF adduct, [(THF)NaP(Si^*i*^Pr_3_)_2_]_2_ (**1a**), were obtained by crystallization of **1** from benzene and THF at room temperature for 4 weeks (see Figure S2). ^1^H NMR (THF-*d*_8_, 400 MHz, 25 °C): δ 3.63–3.60 (m, 6H, THF C*H*_2_ (2,5)), 1.79–1.77 (m, 6H, THF C*H*_2_ (3,4)), 1.12–1.10 (m, 84H, ^*i*^Pr (C*H*_3_)_2_ and ^*i*^Pr (C*H*)). N.B. The integrals corresponding to bound THF in 1a are smaller than expected due to displacement by THF-*d*_8_. ^13^C{^1^H} NMR (THF-*d*_8_, 101 MHz, 25 °C): δ 20.0 (d, ^3^*J*_C–P_ = 3.4 Hz, ^*i*^Pr (*C*H_3_)_2_), 16.3 (d, ^2^*J*_C–P_ = 9.9 Hz, ^*i*^Pr (*C*H)). ^31^P NMR (THF-*d*_8_, 162 MHz, 25 °C): δ – 384.2 (s). ^31^P{^1^H} NMR (THF-*d*_8_, 162 MHz, 25 °C): δ – 384.2 (s). Elemental analysis: calculated for C_44_H_100_Na_2_O_2_P_2_Si_4_ (880.61 g mol^–1^): C 59.95; H 11.43; N 0.00%. Found: C 59.93; H 11.48; N 0.26%.

### General Procedures for the Synthesis of Complexes **2**–**4**

Under an argon atmosphere, the corresponding group 12 halide, MX_2_ (MX_2_ = ZnCl_2_, CdI_2_, HgBr_2_) (0.227 mmol), and the sodium salt **1** (200 mg, 0.542 mmol) were suspended in diethyl ether (10 mL) and stirred at room temperature for 16 h. Diethyl ether was removed under reduced pressure, producing a white solid. To this, *n*-hexane (10 mL) was added, and the white suspension was filtered. The solvent of the colorless filtrate was removed under reduced pressure yielding the crude products. Crystals suitable for single-crystal X-ray diffraction were grown by recrystallization from *n*-hexane at −30 °C.

#### Data for Zn[P(Si^*i*^Pr_3_)_2_]_2_ (**2**)

Colorless crystals (69.7 mg, 0.092 mmol, 41%). ^1^H NMR (C_6_D_6_, 400 MHz, 25 °C): δ 1.29 (m, 84H, ^*i*^Pr (C*H*_3_)_2_ and ^*i*^Pr (C*H*)). ^13^C{^1^H} NMR (C_6_D_6_, 101 MHz, 25 °C): δ 19.8 (m, ^3^*J*_CP_ = 3.6 Hz, ^*i*^Pr (*C*H_3_)_2_), 16.5 (m, ^2^*J*_CP_ = 10.7 Hz, ^*i*^Pr (*C*H)). ^29^Si{^1^H} NMR (C_6_D_6_ 99 MHz, 25 °C): δ 24.7 (d, ^1^*J*_Si–P_ = 37.5 Hz). ^31^P{^1^H} NMR (C_6_D_6_, 162 MHz, 25 °C): δ – 287.8 (s). ATR-FTIR ν_max_ (cm^–1^) 2941 (br), 2860 (s), 2811 (w), 1460 (s), 1379 (m), 1363 (m), 1224 (w), 1068 (m), 1013 (s), 986 (s), 916 (w), 877 (s), 657 (s), 630 (s), 555 (s), 507 (s). HRMS (APCI), *m*/*z*: [M + H]^+^ calculated for C_36_H_85_ZnP_2_Si_4_: 755.4490, found: 755.4523 (error = 4.5 ppm). Elemental analysis: calculated for C_36_H_84_ZnP_2_Si_4_ (754.44 g mol^–1^): C 57.14; H 11.19; N 0.00%. Found: C 56.63; H 11.18; N 0.20%.

A second polymorph of **2**, referred to as **2a**, was crystallized by slow evaporation from diethyl ether and was also characterized by single-crystal X-ray diffraction.

#### Cd[P(Si^*i*^Pr_3_)_2_]_2_ (**3**)

Colorless crystals (52.3 mg, 0.065 mmol, 57%). ^1^H NMR (C_6_D_6_, 400 MHz, 25 °C): δ 1.29–1.28 (m, 84H, ^*i*^Pr (C*H*_3_)_2_ and ^*i*^Pr (C*H*)). ^13^C{^1^H} NMR (C_6_D_6_, 101 MHz, 25 °C): δ 19.8 (m, ^3^*J*_CP_ = 3.7 Hz, ^*i*^Pr (*C*H_3_)_2_), 16.7 (m, ^2^*J*_CP_ = 10.3 Hz, ^*i*^Pr (*C*H)). ^29^Si{^1^H} NMR (C_6_D_6_, 99 MHz, 25 °C): δ 24.4 (d, ^1^*J*_SiP_ = 48.2 Hz). ^31^P{^1^H} NMR (C_6_D_6_, 162 MHz, 25 °C): δ – 284.0 (s). ^113^Cd NMR (C_6_D_6_, 89 MHz, 25 °C): δ 137.68 (t, ^1^*J*_CdP_ = 350 Hz). ATR-FTIR ν_max_ (cm^–1^) 2942 (br), 2862 (s), 2819 (w), 1459 (s), 1379 (m), 1363 (m), 1226 (w), 1069 (m), 1013 (s), 992 (s), 917 (w), 878 (s), 658 (s), 630 (s), 568 (s), 505 (s), 481 (s).

#### Hg[P(Si^*i*^Pr_3_)_2_]_2_ (**4**)

Colorless crystals (67.6 mg, 0.076 mmol, 55%). ^1^H NMR (C_6_D_6_, 500 MHz, 25 °C): δ 1.30 (s, 84H, ^*i*^Pr (C*H*_3_)_2_ and ^*i*^Pr (C*H*)). ^13^C{^1^H} NMR (C_6_D_6_, 126 MHz, 25 °C): δ 19.7 (m, ^3^*J*_CP_ = 3.5 Hz, ^*i*^Pr (*C*H_3_)_2_), 16.7 (m, ^2^*J*_CP_ = 10.2 Hz, ^*i*^Pr (*C*H)). ^29^Si{^1^H} NMR (C_6_D_6_, 99 MHz, 25 °C): δ 25.3 (m). ^31^P{^1^H} NMR (C_6_D_6_, 99 MHz, 25 °C): δ – 209.2 (s). ^199^Hg NMR (C_6_D_6_, 90 MHz, 25 °C): δ 13.2 (t, ^1^*J*_HgP_ = 407.7 Hz). ATR-FTIR ν_max_ (cm^–1^) 2941 (br), 2862 (s), 1457 (s), 1379 (m), 1363 (m), 1226 (w), 1069 (m), 1015 (s), 988 (s), 917 (w), 878 (s), 659 (s), 630 (s), 571 (s), 534 (s), 507 (s), 485 (s).

### Computational Methodology

Geometry optimizations were performed for the models **2**–**4** using coordinates derived from their X-ray crystal structures. These models were geometry-optimized without restraints using the ORCA 5.0.4 software package^94^ utilizing the PBE0 density functional^81,82^ and all-electron ZORA-corrected^83^ def2-TZVP basis sets^84−87^ for all atoms (except Cd and Hg), SARC-ZORA-TZVP^88^ basis sets for the Cd and Hg atoms, along with SARC/J auxiliary basis sets decontracted def2/J up to Kr^95^ and SARC auxiliary basis sets beyond Kr.^88,96−98^ Dispersion corrections were performed with Grimme’s third-generation dispersion correction.^89,90^ TightSCF and TightOpt convergence criteria were employed, and the location of true minima in these optimizations was confirmed by frequency analysis, which demonstrated that no imaginary vibrations were present.

Geometry optimizations for the linear analogues **2’**, **3′**, and **4’** were performed using coordinates derived from the X-ray crystal structure of Cd[P(SiPh_3_)_2_]_2_.^54^ These optimizations were carried out using the same methodology described above but with the P–M–P (M = Zn, Cd, Hg) angle constrained to 180°.

Two geometry optimizations were performed for Cd[P(SiMe_3_)_2_]_2_, using coordinates derived from either the optimized structure of **2** (referred to as “bent” Cd[P(SiMe_3_)_2_]_2_) or the optimized structure of 2’ (referred to as “linear” Cd[P(SiMe_3_)_2_]_2_). The optimizations were conducted without restraints by using the same methodology described above. The “bent” and “linear” structures optimized to P–Cd–P bond angles of 177.5° and 179.9°, respectively, with the “bent” structure being Δ*G* = −0.1 kcal mol^–1^ more stable.

## Supporting Information Available

Additional experimental details, materials and methods, HRMS spectra, IR spectra, NMR spectra, DFT calculations, and crystal data (PDF)
xyz files (ZIP)
