# Supplementary material for: Synthesis of the Bulky Phosphanide [P(SiiPr3)2]− and Its Stabilization of Low-Coordinate Group 12 Complexes
Source: Inorg Chem. 2024 Oct 10;63(43):20286–94. doi: 10.1021/acs.inorgchem.4c03134 (PMC11523236; doi:10.1021/acs.inorgchem.4c03134)
Supplement: Supplementary file 1 — ic4c03134_si_001.pdf [file ic4c03134_si_001.pdf]

Supplementary Information For:

Synthesis of the Bulky Phosphanide $[P(Si^{i}Pr_3)_2]^{-}$ and its Stabilization of Low-Coordinate Group 12 Complexes.

*Olivia P. Churchill,[a], Antonia Dase,[a] Laurence J. Taylor,[a] Stephen P. Argent,[a] Nathan T. Coles,[a] Gavin S. Walker[c] and Deborah L. Kays[a,b],**

[a] School of Chemistry, University Park, University of Nottingham, Nottingham, NG7 2RD (UK)

[b] School of Chemistry, Cardiff University, Main Building, Cardiff, CF10 3AT (UK)

[c] Advanced Materials Research Group, Faculty of Engineering, University of Nottingham, Nottingham, NG7 2GA (UK)

* Email: KaysD@Cardiff.ac.uk

Contents

S1 – Crystallography 3

S1.1 – Crystallography Experimental 3

S1.2 – Supplementary Crystallographic Figures 3

S1.3 – Crystallography Data 4

S1.4 – Disorder Modelling 5

S1.4.1 – [(THF)NaP(SiiPr3)2]2 (1a) 5

S1.4.2 – $Cd[P(Si^iPr_3)_2]_2$ (**3**) 5

S1.4.3 – $Hg[P(Si^iPr_3)_2]_2$ (**4**) 5

S2 – NMR Spectra 6

S2.1 – $NaP(Si^iPr_3)_2$ (**1**) 6

S2.2 – $[(THF)NaP(Si^iPr_3)_2]_2$ (**1a**) 8

S2.3 – $Zn[P(Si^iPr_3)_2]_2$ (**2**) 10

S2.4 – $Cd[P(Si^iPr_3)_2]_2$ (**3**) 12

S2.5 – $Hg[P(Si^iPr_3)_2]_2$ (**4**) 15

S3 – IR Spectra 18

S3.1 – $NaP(Si^iPr_3)_2$ (**1**) 18

S3.2 – $Zn[P(Si^iPr_3)_2]_2$ (**2**) 19

S3.3 – $Cd[P(Si^iPr_3)_2]_2$ (**3**) 19

S3.4 – $Hg[P(Si^iPr_3)_2]_2$ (**4**) 20

S4 – Mass Spectrometry Data 20

S4.1 – $Zn[P(Si^iPr_3)_2]_2$ (**2**) 20

S5 – Computational Methodology 21

S5.1 – Geometry Optimizations 21

S5.2 – Geometry Optimized Coordinates 21

S5.2.1 – Coordinates for **2** 21

S5.2.2 – Coordinates for **3** 24

S5.2.3 – Coordinates for **4** 27

S5.2.4 – Coordinates for **2’** 30

S5.2.5 – Coordinates for **3’** 32

S5.2.6 – Coordinates for **4’** 35

S5.2.7 – Coordinates for “bent” $Cd[P(SiMe_3)_2]_2$ 38

S5.2.8 – Coordinates for “linear” $Cd[P(SiMe_3)_2]_2$ 39

S6 – References 40

S1 – Crystallography

S1.1 – Crystallography Experimental

Suitable single crystals of **1a**, **2**, **2a**, **3**, and **4** were selected under the protection of Fomblin® (YR-1800 perfluoropolyether oil) then mounted on a polymer-tipped MicroMount™ and rapidly cooled with a stream of N_2 at 120 K. The data were collected on an Oxford Diffraction GV1000 (TitanS2 CCD area detector, mirror-monochromated Cu-Kα radiation source; λ = 1.54184 Å, ω scans), an Oxford Diffraction GV1000 (AtlasS2 CCD area detector, mirror-monochromated Cu-Kα radiation source; λ = 1.54184 Å, ω scans). Data collection was handled by CrysAlisPRO[1] (Rigaku XtaLAB). The collected frames were integrated using CrysAlisPro (Rigaku XtaLAB) and the data were corrected for absorption effects using a Gaussian numerical method with beam profile correction (CrysAlisPro). Structures were solved within Olex2[2] by dual space iterative methods (SHELXT)[3] and all non-hydrogen atoms refined by full-matrix least-squares on all unique F2 values with anisotropic displacement parameters (SHELXL).[4] Hydrogen atoms were refined with constrained geometries and riding thermal parameters. Structures were checked with checkCIF.[5]

S1.2 – Supplementary Crystallographic Figures

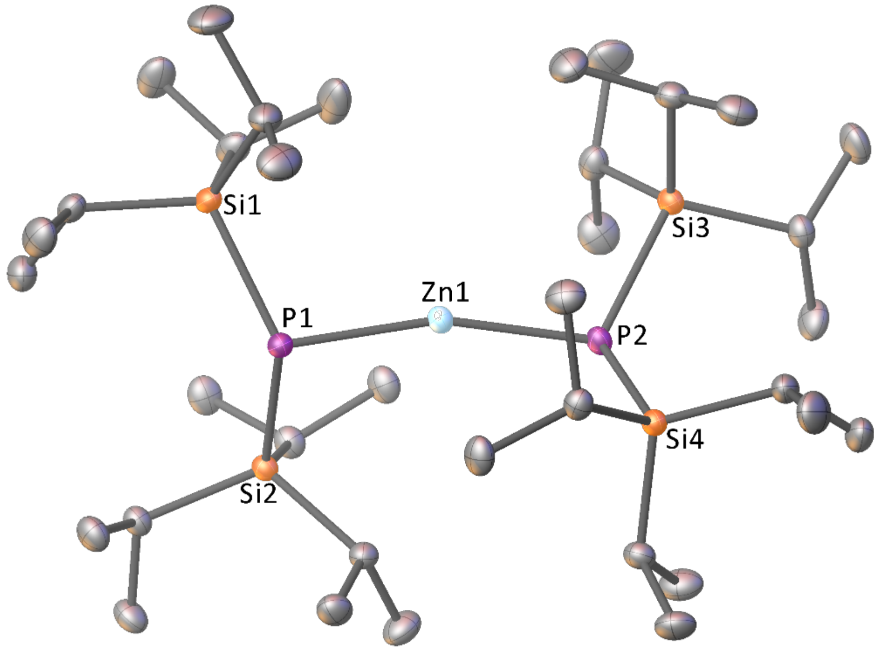

Figure S1: Single Crystal XRD structure of **2a**. Hydrogen atoms omitted for clarity; thermal ellipsoids set at 50% probability.

S1.3 – Crystallography Data

Table S1: Crystallographic data for all X-ray structures presented in this manuscript.

Compound	**(THF)NaP(Si^{i}Pr$_3$)$_2$**	**2**	**2a**	**3**	**4**
Empirical formula	$C_{44}H_{100}Na_2O_2P_2Si_4$	$C_{36}H_{84}P_2Si_4Zn$	$C_{36}H_{84}P_2Si_4Zn$	$C_{36}H_{84}CdP_2Si_4$	$C_{36}H_{84}HgP_2Si_4$
Formula weight	881.51	756.7	756.7	803.73	891.92
Temperature/K	120(2)	120(2)	120(2)	120(2)	120(2)
Crystal system	monoclinic	triclinic	triclinic	triclinic	triclinic
Space group	C2/c	P-1	P-1	P-1	P-1
a/Å	13.14520(10)	11.3873(2)	11.2588(3)	12.1217(3)	12.1286(2)
b/Å	18.1813(2)	11.7617(3)	13.8675(3)	14.3471(3)	14.3407(3)
c/Å	23.3491(2)	19.9413(3)	16.3177(4)	15.5450(4)	15.5091(3)
α/°	90	96.533(2)	72.225(2)	66.496(2)	66.948(2)
β/°	98.4840(10)	95.5770(10)	80.702(2)	80.225(2)	80.2650(10)
γ/°	90	118.925(2)	68.167(2)	66.229(2)	66.140(2)
Volume/Å^3	5519.29(9)	2286.60(9)	2248.74(10)	2268.70(10)	2269.82(9)
Z	4	2	2	2	2
ρ_{calc}g/cm^3	1.061	1.099	1.118	1.177	1.305
μ/mm^{-1}	1.924	2.556	2.599	5.68	7.906
F(000)	1952.0	832	832	868	932
Crystal size/mm^3	0.34 × 0.21 × 0.17	0.26 × 0.17 × 0.12	0.18 × 0.13 × 0.05	0.269 × 0.217 × 0.104	0.179 × 0.132 × 0.066
Radiation	Cu Kα (λ = 1.54184)	Cu Kα (λ = 1.54184)	Cu Kα (λ = 1.54184)	Cu Kα (λ = 1.54184)	Cu Kα (λ = 1.54184)
2Θ range for data collection/°	7.656 to 144.898	8.74 to 145.31	7.128 to 146.552	7.236 to 148.026	7.22 to 145.67
Index ranges	-16 ≤ h ≤ 16, -22 ≤ k ≤ 22, -28 ≤ l ≤ 28	-14 ≤ h ≤ 14, -14 ≤ k ≤ 14, -24 ≤ l ≤ 24	-13 ≤ h ≤ 13, -17 ≤ k ≤ 17, -20 ≤ l ≤ 19	-14 ≤ h ≤ 15, -17 ≤ k ≤ 17, -19 ≤ l ≤ 19	-15 ≤ h ≤ 15, -17 ≤ k ≤ 17, -19 ≤ l ≤ 19
Reflections collected	47498	40249	38932	33280	62580
Independent reflections	5457 [R_{int} = 0.0210, R_{sigma} = 0.0097]	8976 [R_{int} = 0.0255, R_{sigma} = 0.0193]	8879 [R_{int} = 0.0357, R_{sigma} = 0.0236]	8770 [R_{int} = 0.0323, R_{sigma} = 0.0273]	8938 [R_{int} = 0.0385, R_{sigma} = 0.0236]
Data/restraints/parameters	5457/457/310	8976/0/412	8879/0/412	8770/884/508	8938/1801/556
Goodness-of-fit on F^2	1.054	1.056	1.026	1.014	1.024
Final R indexes [I>=2σ (I)]	R_1 = 0.0499, wR_2 = 0.1271	R_1 = 0.0223, wR_2 = 0.0572	R_1 = 0.0230, wR_2 = 0.0581	R_1 = 0.0244, wR_2 = 0.0600	R_1 = 0.0183, wR_2 = 0.0453
Final R indexes [all data]	R_1 = 0.0504, wR_2 = 0.1275	R_1 = 0.0245, wR_2 = 0.0584	R_1 = 0.0258, wR_2 = 0.0596	R_1 = 0.0268, wR_2 = 0.0614	R_1 = 0.0189, wR_2 = 0.0457
Largest diff. peak/hole / e Å^{-3}	1.14/-0.72	0.40/-0.18	0.32/-0.32	0.61/-0.51	0.96/-1.27

S1.4 – Disorder Modelling

S1.4.1 – [(THF)NaP(Si^{i}Pr$_3$)$_2$]$_2$ (**1a**)

The sodium bound tetrahydrofuran (THF) solvent residue is disordered over three overlapping conformations with similar oxygen positions. The occupancies of the three components were refined and restrained to sum to unity with a linear restraint (SUMP) resulting in values of 0.27(1), 0.25(1) and 0.48(1) for residues A, B and C respectively. The geometries of the three THF residues are constrained to refine as rigid bodies with coordinates taken from Ilia's Idealized Molecular Geometry Library (https://xray.chem.wisc.edu/idealized-molecular-geometry-library/). The geometries of residues A and B are fixed in 'envelope' Cs whilst the geometry of residue C is fixed in a 'twisted' C2 conformation. Rigid bond, similarity and isotropic character restraints were applied to the anisotropic displacement parameters of all the disordered atoms (RIGU, SIMU, ISOR). The anisotropic displacement parameters of the three closely overlapping oxygen atoms were constrained to be identical (EADP). All hydrogen atoms were geometrically placed before being refined with a riding model. A short contact of 2.612 Å between sodium Na1 and methyl hydrogen H3C has been excluded from the connectivity list and is assumed to be a consequence of the limitations of the riding hydrogen model.

S1.4.2 – Cd[P(Si^{i}Pr$_3$)$_2$]$_2$ (**3**)

Conformational disorder was modelled for isopropyl moieties C21b, C21d, C31b and C31d; their occupancies were refined and for each pair constrained to sum to unity resulting in values for the major component of 0.52(1), 0.52(2), 0.50(1) and 0.51(1) respectively. The geometries of all isopropyl moieties in the structure were restrained to have similar 1,2 and 1,3 bond and angle distances (SADI). Rigid bond and similarity restraints were applied to the anisotropic displacement parameters for all disordered atoms in the structure (RIGU, SIMU). The anisotropic displacement parameters of closely overlapping atoms from the disorder components were constrained to be identical (EADP). The anisotropic displacement parameters of disordered carbon atoms C31d and C31w were restrained to have more isotropic character (ISOR).

S1.4.3 – Hg[P(Si^{i}Pr$_3$)$_2$]$_2$ (**4**)

Disorder is modelled for two silicon-triisopropyl moieties and one isopropyl moiety. For all three disorder component pairs the occupancies were refined and constrained to sum to unity resulting in values of 0.50(1), 0.51(1) and 0.55(2) for the major occupancy components of silicon-triisopropyl moiety Si1, silicon-triisopropyl moiety Si4 and isopropyl moiety C31C respectively. All chemically similar 1,2 and 1,3 distances amongst the disorder and not disordered silicon-triisopropyl moieties were restrained to be similar (SADI). Rigid bond and similarity restraints were applied to the anisotropic displacement parameters of all isopropyl moieties and disordered atoms (RIGU, SIMU). The anisotropic displacement parameters of all closely overlapping silicon and carbon atoms of disorder components were constrained to be identical (EADP).

S2 – NMR Spectra

S2.1 – NaP(Si^{i}Pr$_3$)$_2$ (**1**)

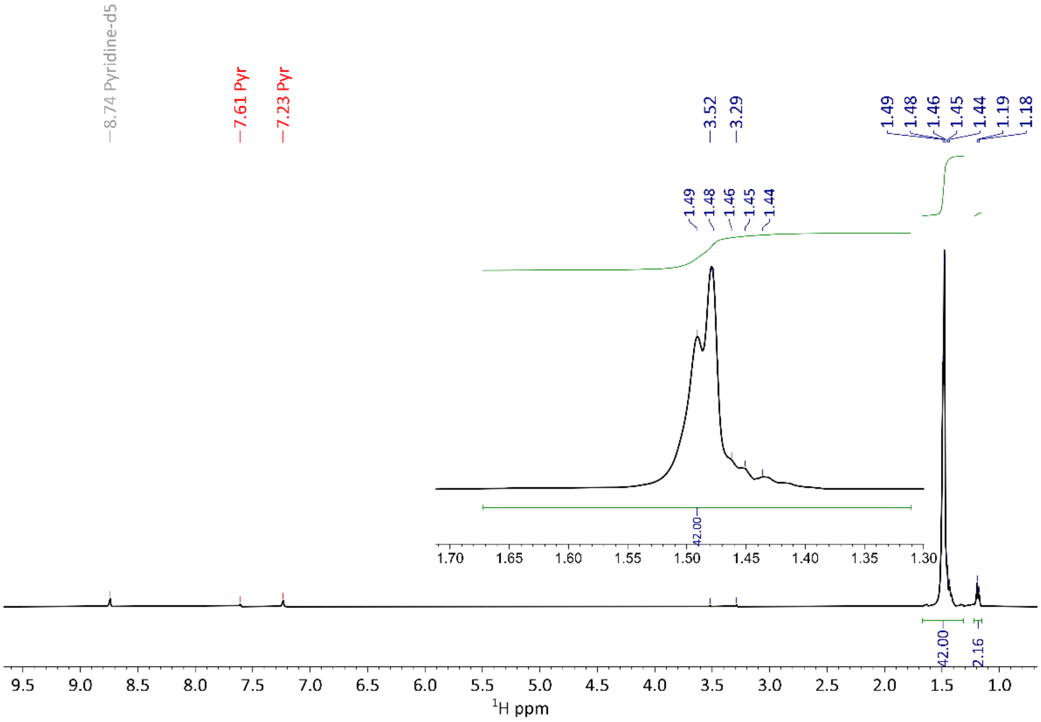

Figure S2 ^{1}H NMR (400 MHz, 25 °C) spectrum for NaP(Si^{i}Pr$_3$)$_2$ **1** in pyridine-d$_5$. Minor signals at δ_H = 1.19 and 1.18 ppm correspond to trace quantities of (^{i}Pr$_3$Si)$_2$PH and at δ_H = 3.52 and 3.29 correspond to residual DME.

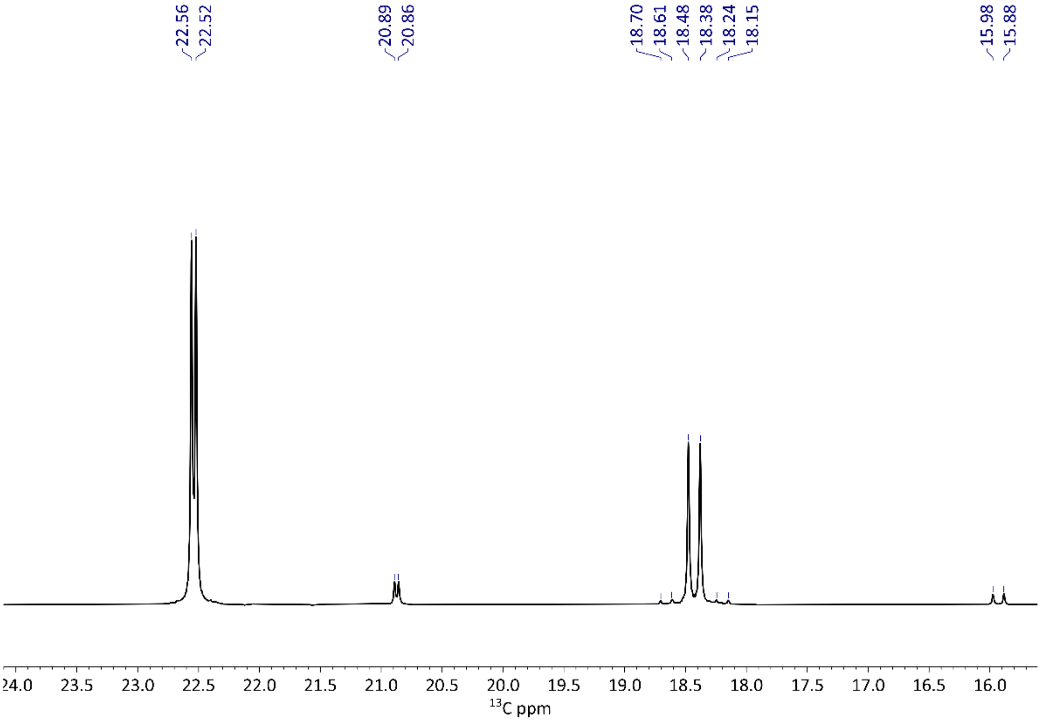

Figure S3 ^{13}C{^{1}H} NMR (101 MHz, 25 °C) spectrum for NaP(Si^{i}Pr$_3$)$_2$ **1** in pyridine-d$_5$. The minor signals at δ_C = 20.88 and 15.93 ppm correspond to trace quantities of (^{i}Pr$_3$Si)$_2$PH.

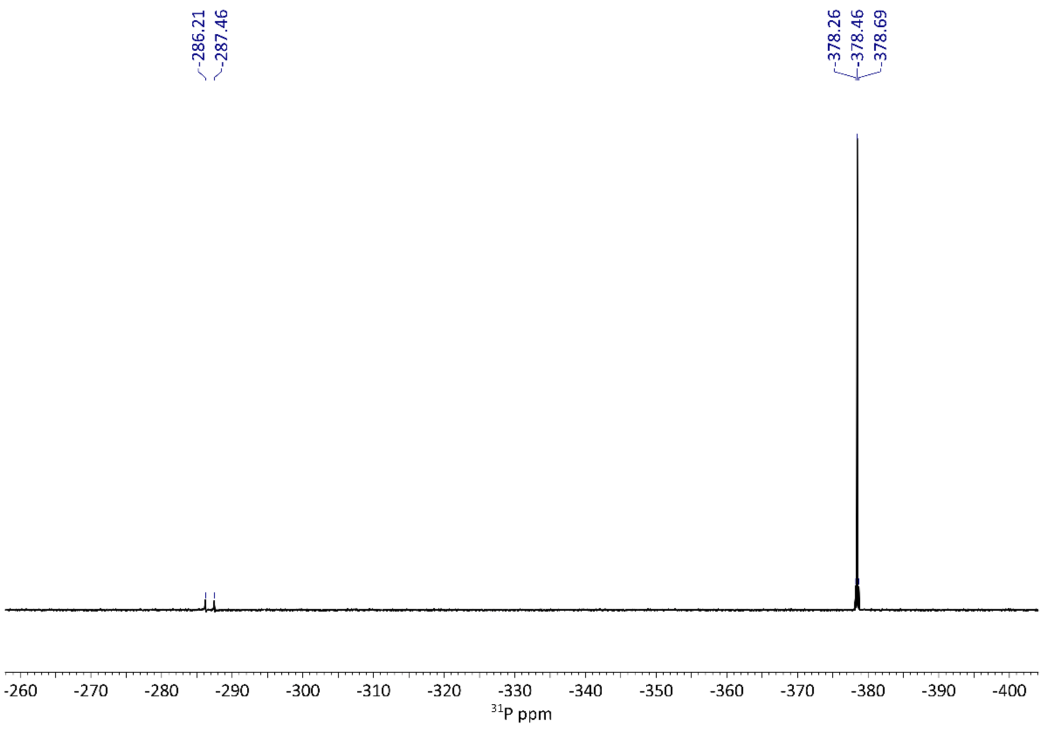

Figure S4 ^{31}P NMR (162 MHz, 25 °C) spectrum for $NaP(Si^iPr_3)_2$ **1** in pyridine-d$_5$. The minor signal at δ_P = −268.84 ppm corresponds to trace quantities of $(^iPr_3Si)_2PH$.

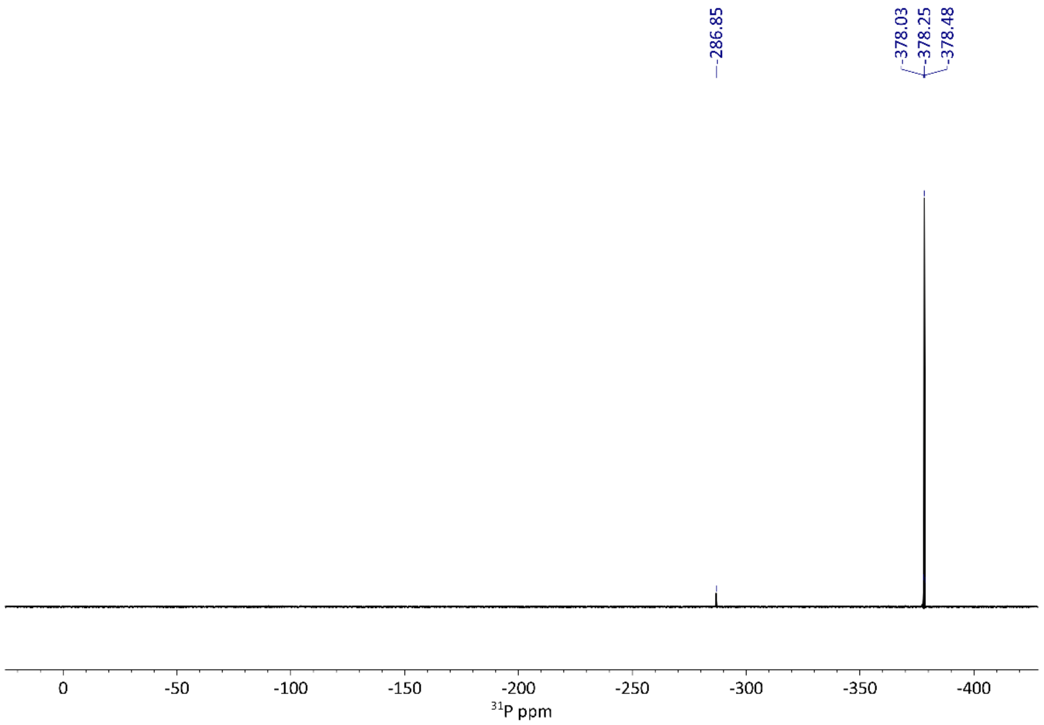

Figure S5 $^{31}P\{^1H\}$ NMR (162 MHz, 25 °C) spectrum for $NaP(Si^iPr_3)_2$ **1** in pyridine-d$_5$. The minor signal at δ_P = −268.9 ppm corresponds to trace quantities of $(^iPr_3Si)_2PH$.

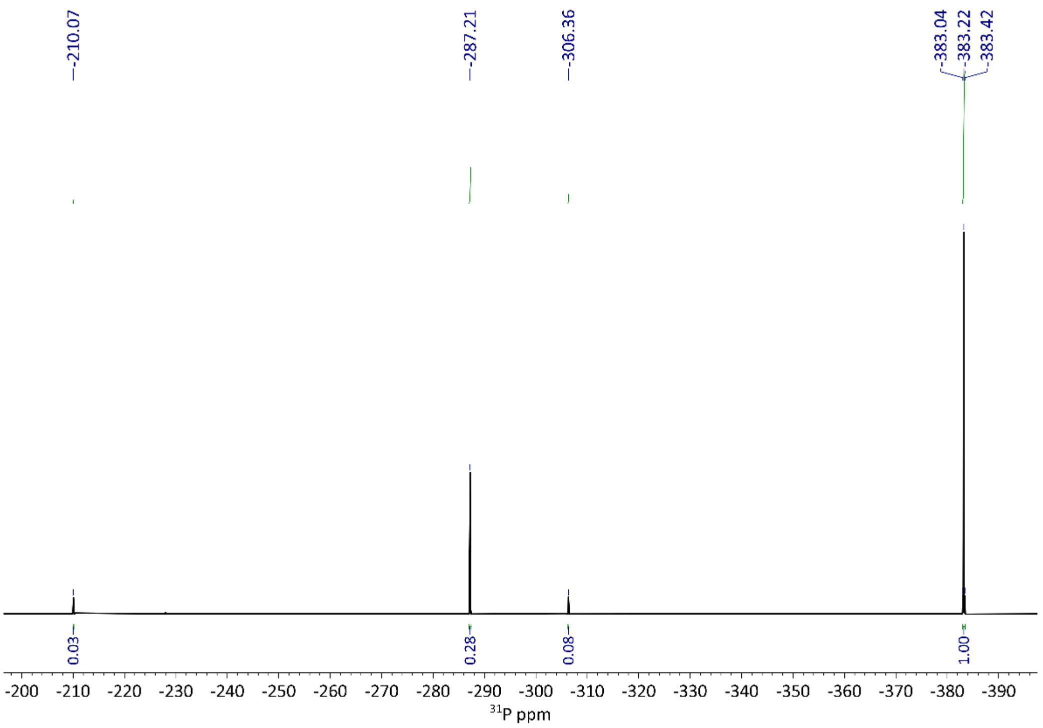

Figure S6 $^{31}P\{^1H\}$ NMR (162 MHz, 25 °C) spectrum of the crude reaction mixture of the synthesis of $NaP(Si^iPr_3)_2$ **1** in non-deuterated DME showing the approximate ratio of by-products formed. The peak at δ_P = −287.2 corresponds to $(^iPr_3Si)_2PH$ and the peak at δ_P −306.4 corresponds to $P(Si^iPr_3)_3$. The peak at δ_P −210.1 is suggested to correspond to a minor phosphorus-containing product.

S2.2 – $[(THF)NaP(Si^iPr_3)_2]_2$ (**1a**)

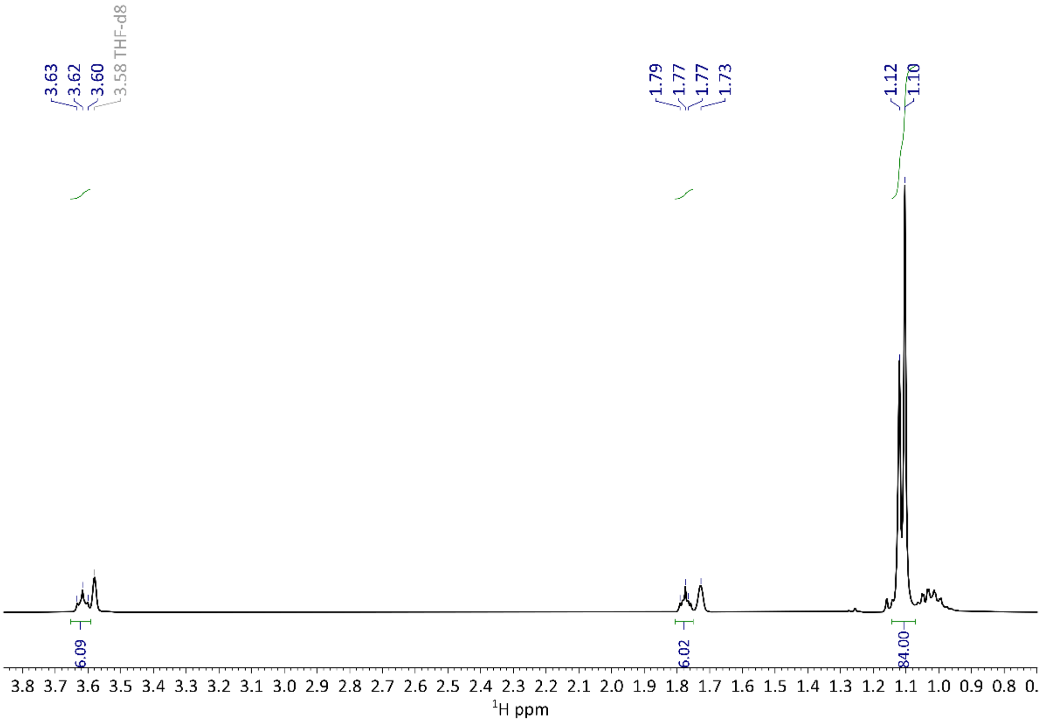

Figure S7 ^1H NMR (400 MHz, 25 °C) spectrum for $[(THF)NaP(Si^iPr_3)_2]_2$ **1a** in THF-d_8. Integrals corresponding to bound THF are smaller than expected due to the displacement by THF-d_8.

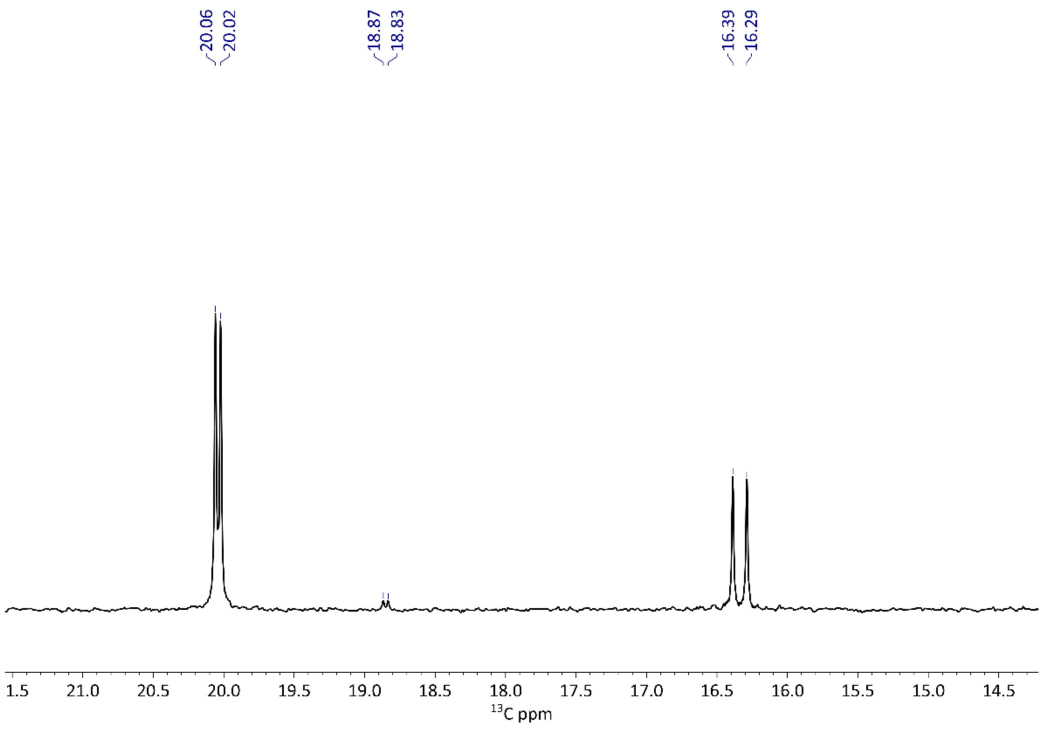

Figure S8 $^{13}C\{^{1}H\}$ NMR (101 MHz, 25 °C) spectrum for $[(THF)NaP(Si^{i}Pr_3)_2]_2$ **1a** in THF-d_8. The minor signal at δ_C = 18.85 ppm corresponds to trace quantities of $(^{i}Pr_3Si)_2PH$.

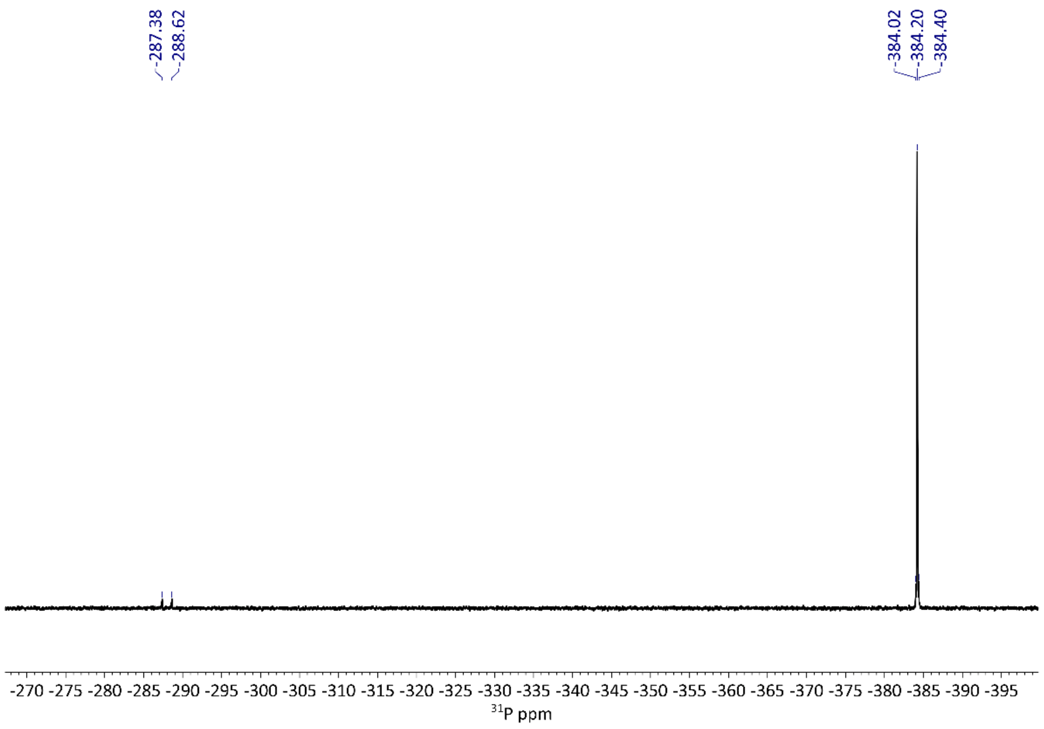

Figure S9 ^{31}P NMR (162 MHz, 25 °C) spectrum for $[(THF)NaP(Si^{i}Pr_3)_2]_2$ **1a** in THF-d_8. The minor signal at δ_P = −287.0 ppm corresponds to trace quantities of $(^{i}Pr_3Si)_2PH$.

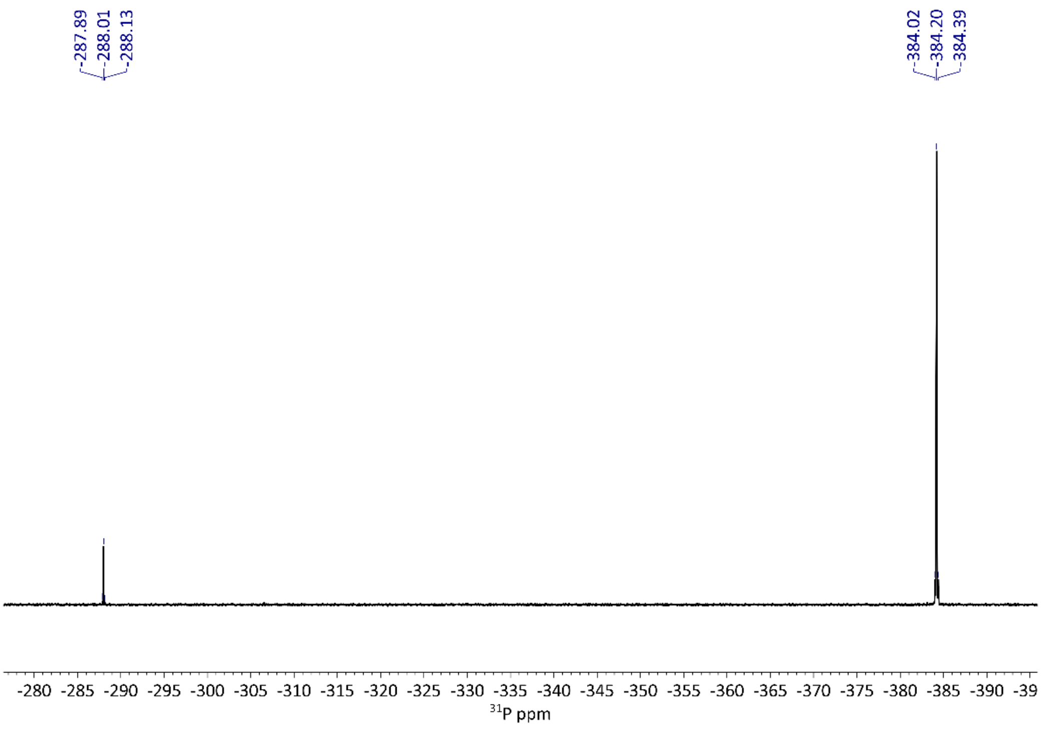

Figure S10 $^{31}P\{^1H\}$ NMR (162 MHz, 25 °C) spectrum for [(THF)NaP(Si^{i}Pr$_3$)$_2$]$_2$ **1a** in THF-d$_8$. The minor signal at δ_P −288.01 ppm corresponds to trace quantities of (^{i}Pr$_3$Si)$_2$PH.

S2.3 – Zn[P(Si^{i}Pr$_3$)$_2$]$_2$ (2)

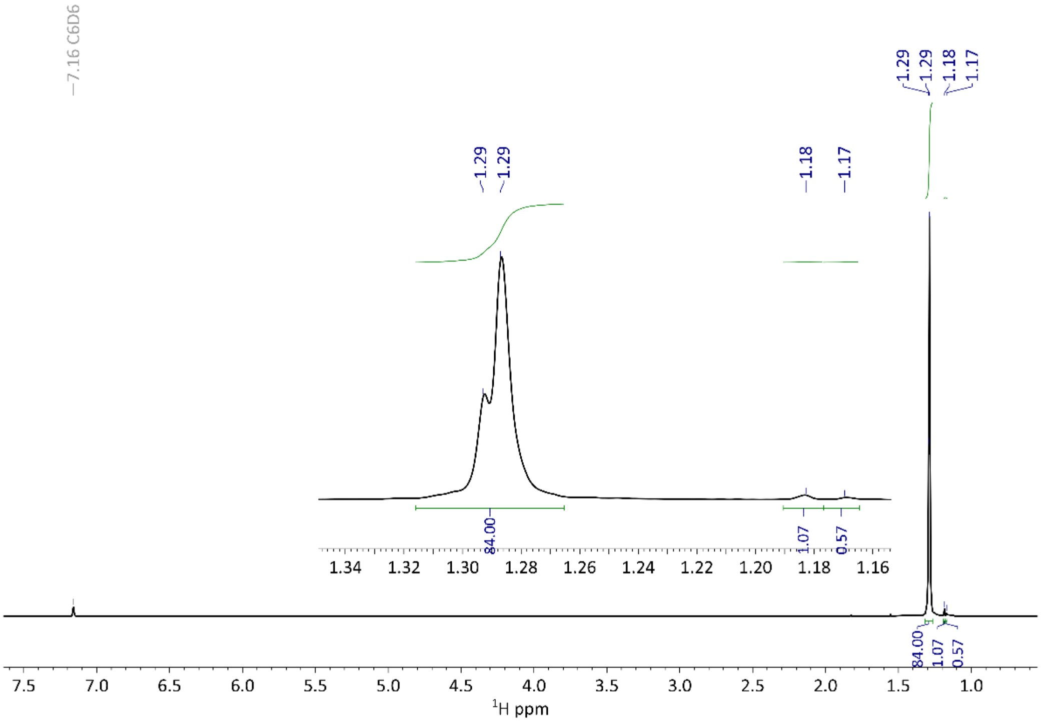

Figure S11 ^1H NMR (400 MHz, 25 °C) spectrum for Zn[P(Si^{i}Pr$_3$)$_2$]$_2$ **2** in C_6D_6. Minor signals at δ_H 1.18 and 1.17 ppm correspond to trace quantities of (^{i}Pr$_3$Si)$_2$PH.

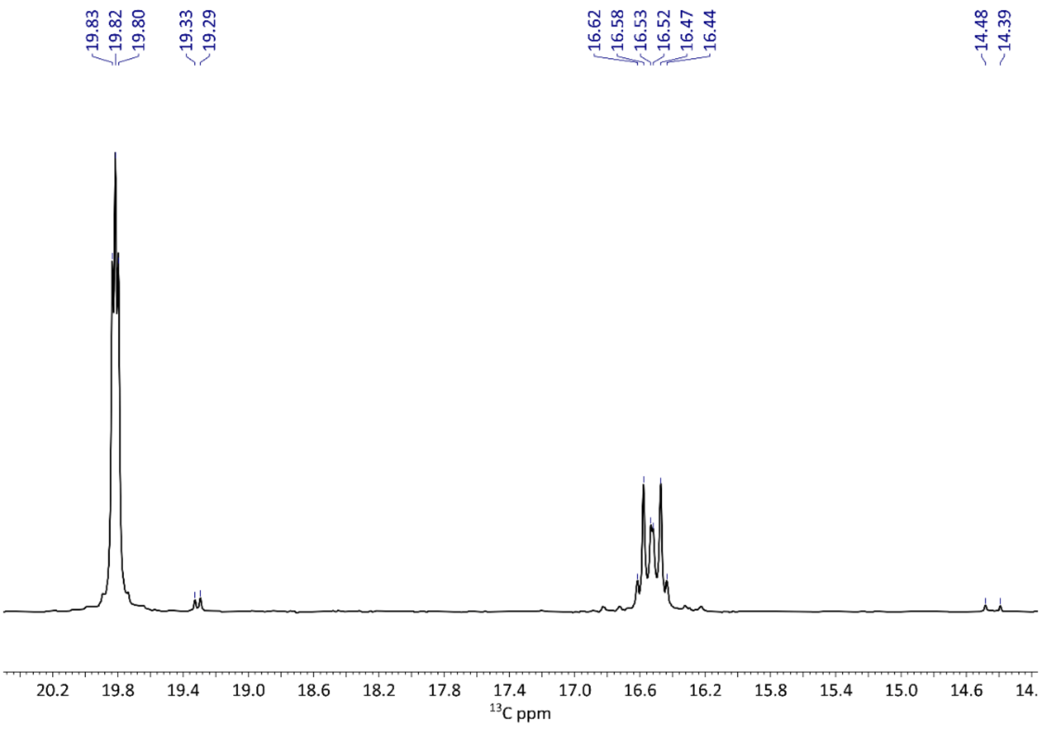

Figure S12 $^{13}C\{^{1}H\}$ NMR (101 MHz, 25 °C) spectrum for $Zn[P(Si^{i}Pr_3)_2]_2$ **2** in C_6D_6. Minor signals at δ_C = 19.6 and 14.4 correspond to trace quantities of $(^{i}Pr_3Si)_2PH$.

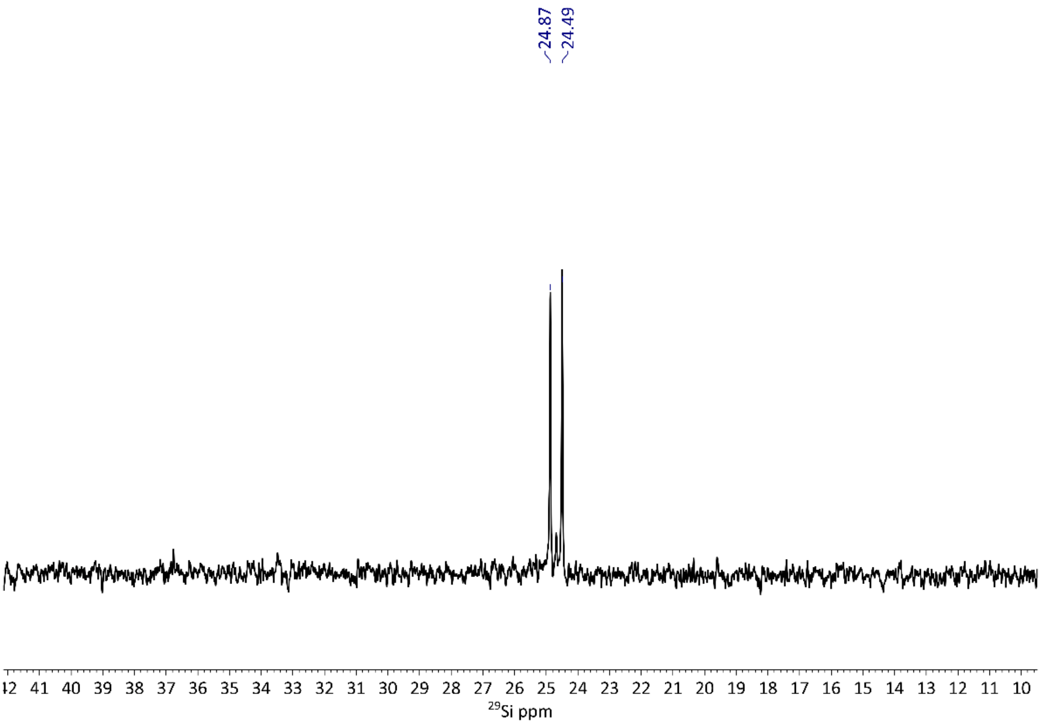

Figure S13 ^{29}Si NMR (99 MHz, 25 °C) spectrum for $Zn[P(Si^{i}Pr_3)_2]_2$ **2** in C_6D_6.

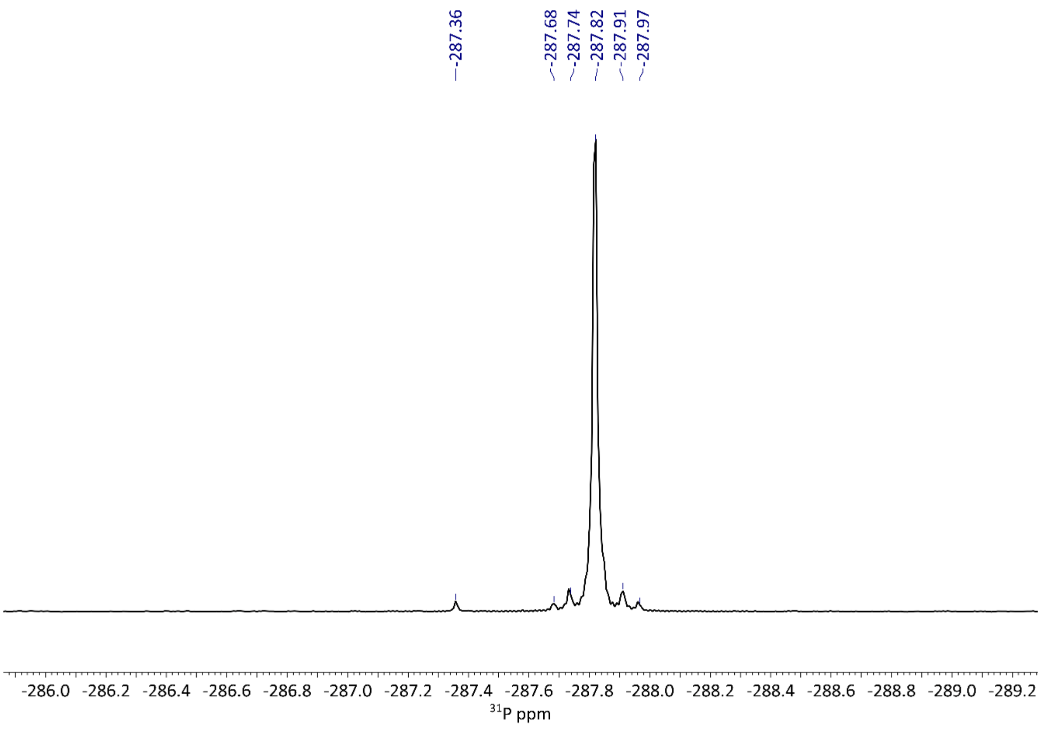

Figure S14 $^{31}P\{^{1}H\}$ NMR (126 MHz, 25 °C) spectrum for $Zn[P(Si^{i}Pr_3)_2]_2$ **2** in C_6D_6. The minor signal at δ_P 287.4 ppm corresponds to trace quantities of $(^{i}Pr_3Si)_2PH$.

S2.4 – $Cd[P(Si^{i}Pr_3)_2]_2$ (**3**)

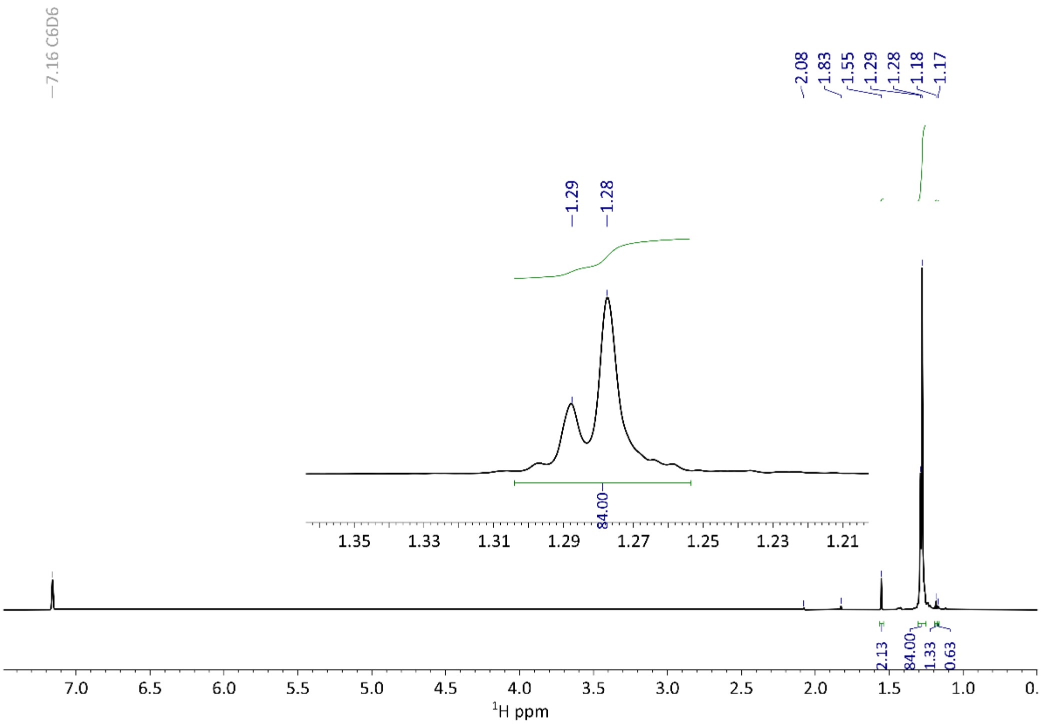

Figure S15 ^{1}H NMR (400 MHz, 25 °C) spectrum for $Cd[P(Si^{i}Pr_3)_2]_2$ **3** in C_6D_6. Minor signals at δ_H = 1.18 and 1.17 ppm correspond to trace quantities of $(^{i}Pr_3Si)_2PH$.

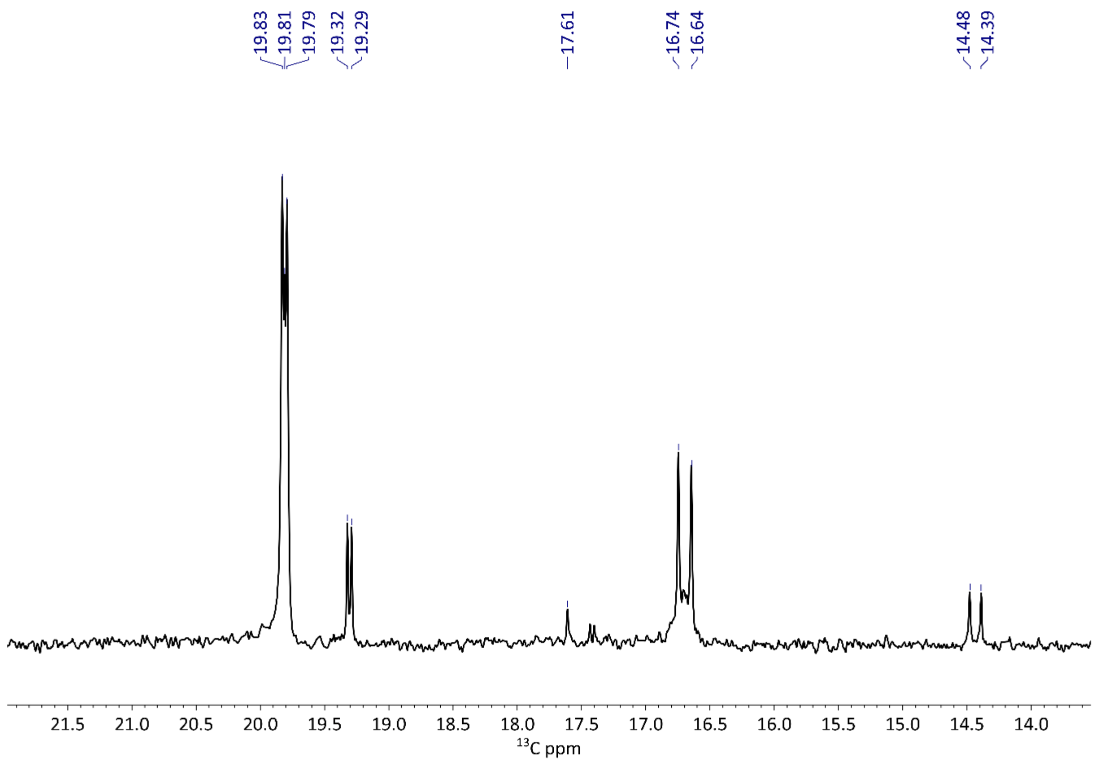

Figure S16 $^{13}C\{^{1}H\}$ NMR (101 MHz, 25 °C) spectrum for Cd[P(Si^{i}Pr$_3$)$_2$]$_2$ **3** in C_6D_6. Minor signals at δ_C = 19.3 and 14.4 ppm correspond to trace quantities of (^{i}Pr$_3$Si)$_2$PH.

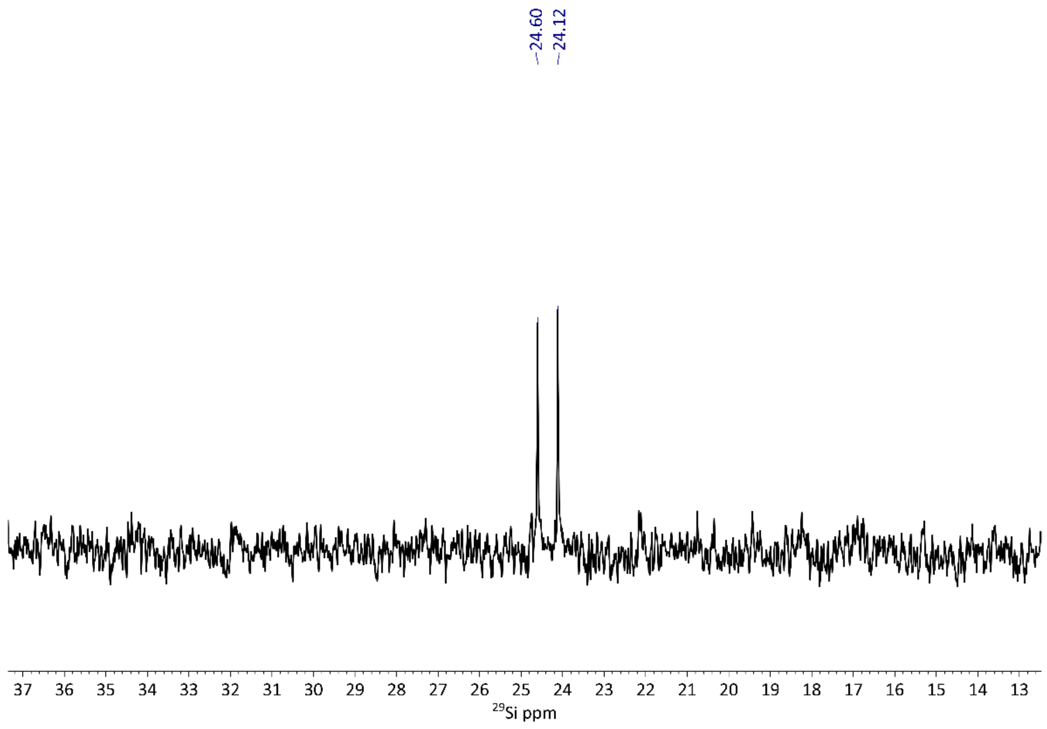

Figure S17 ^{29}Si NMR (99 MHz, 25 °C) spectrum for Cd[P(Si^{i}Pr$_3$)$_2$]$_2$ **2** in C_6D_6.

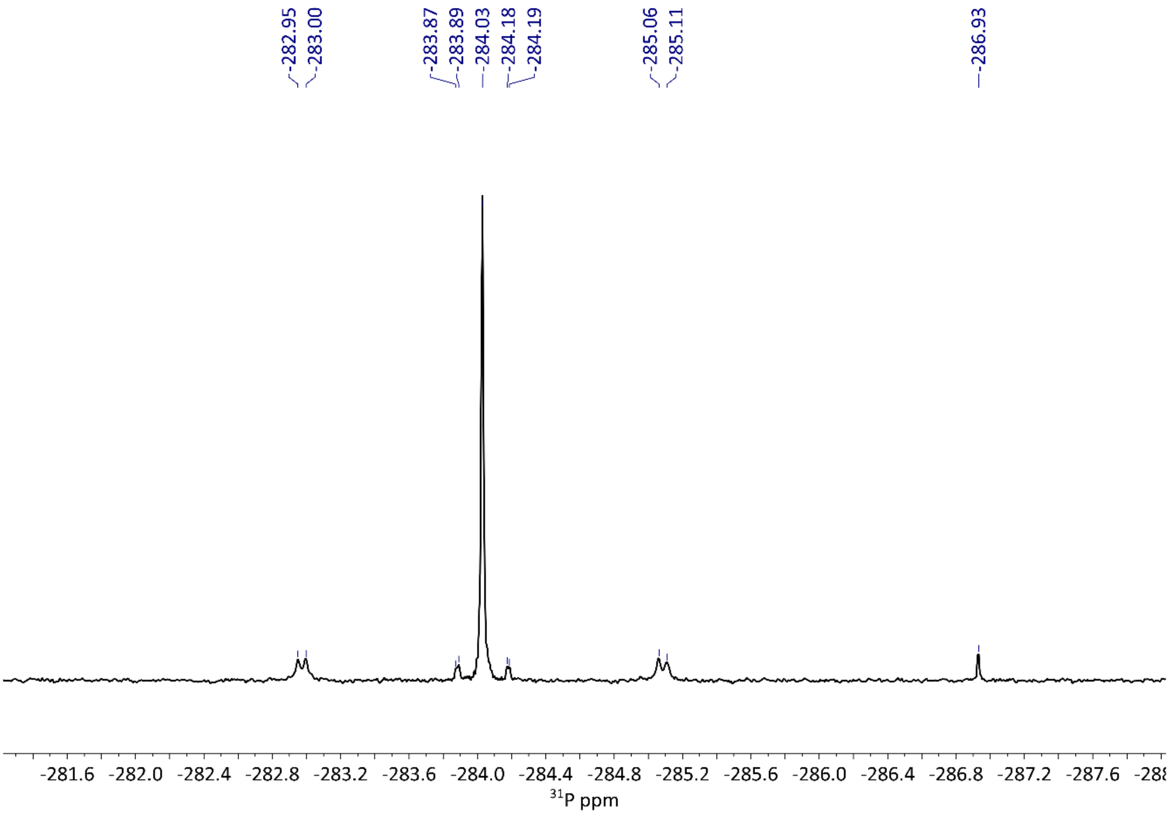

Figure S18 $^{31}P\{^{1}H\}$ NMR (162 MHz, 25 °C) spectrum for $Cd[P(Si^{i}Pr_3)_2]_2$ **3** in C_6D_6. The minor signal at δ_P = −286.9 ppm corresponds to trace quantities of $(^{i}Pr_3Si)_2PH$.

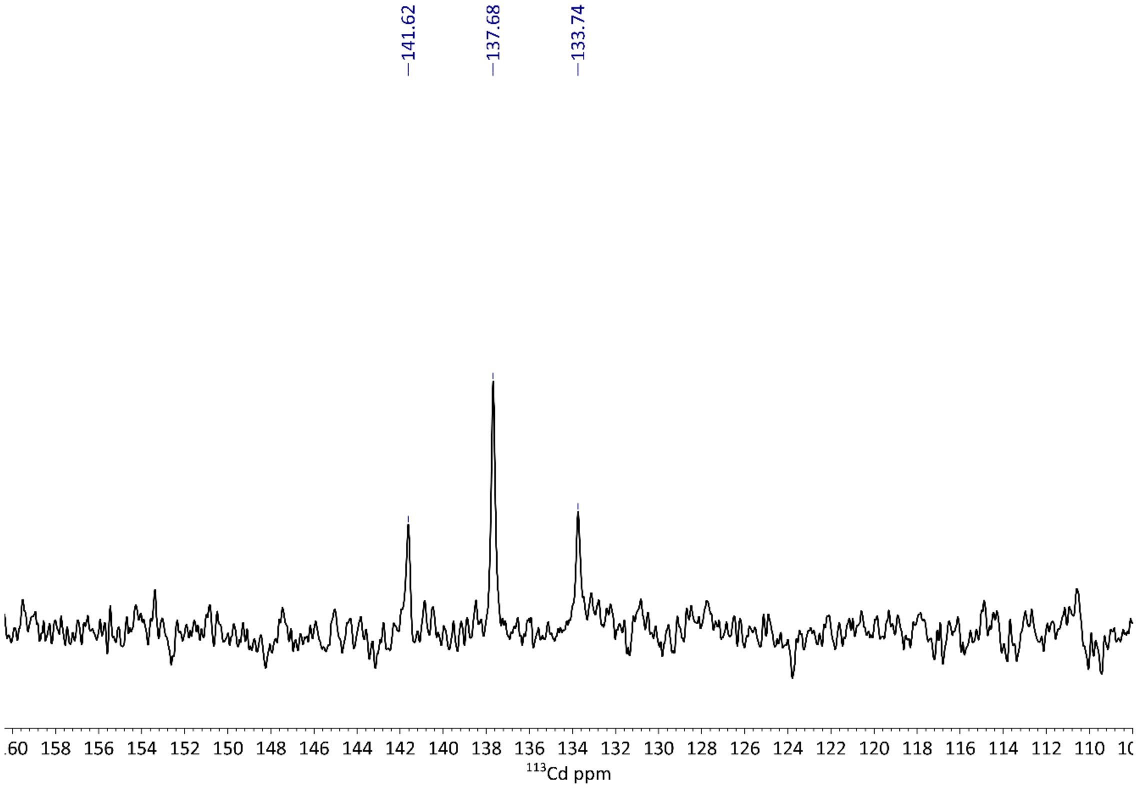

Figure S19 ^{133}Cd NMR (89 MHz, 25 °C) spectrum for $Cd[P(Si^{i}Pr_3)_2]_2$ **3** in C_6D_6.

S2.5 – Hg[P(Si^{i}Pr$_3$)$_2$]$_2$ (**4**)

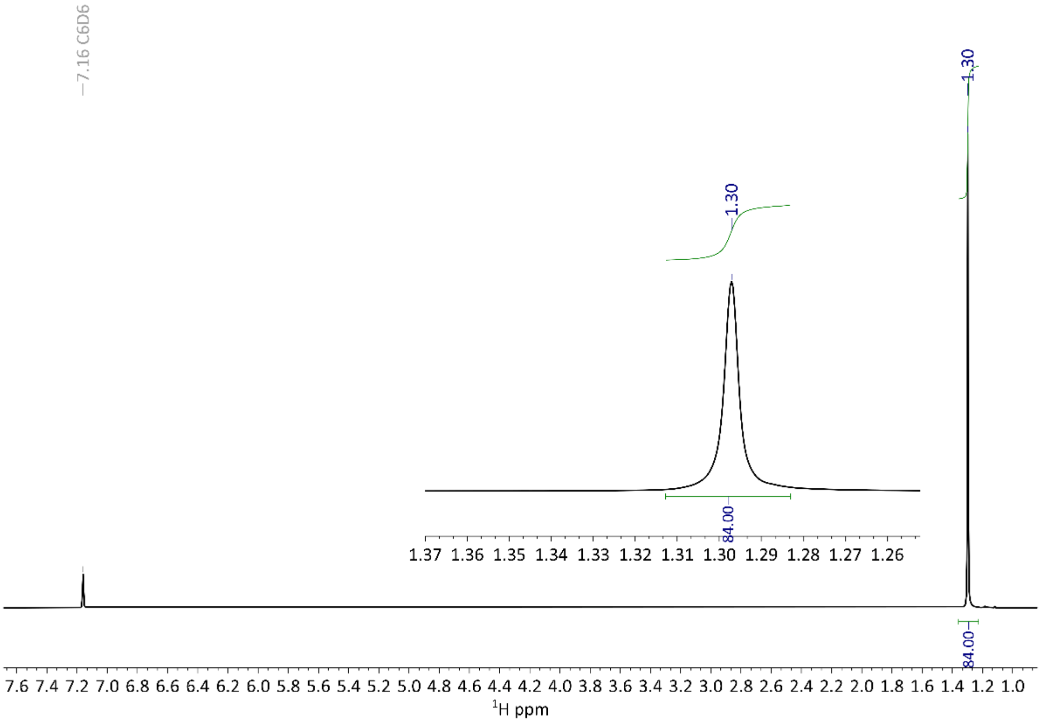

Figure S20 ^{1}H NMR (500 MHz, 25 °C) spectrum for Hg[P(Si^{i}Pr$_3$)$_2$]$_2$ **4** in C_6D_6.

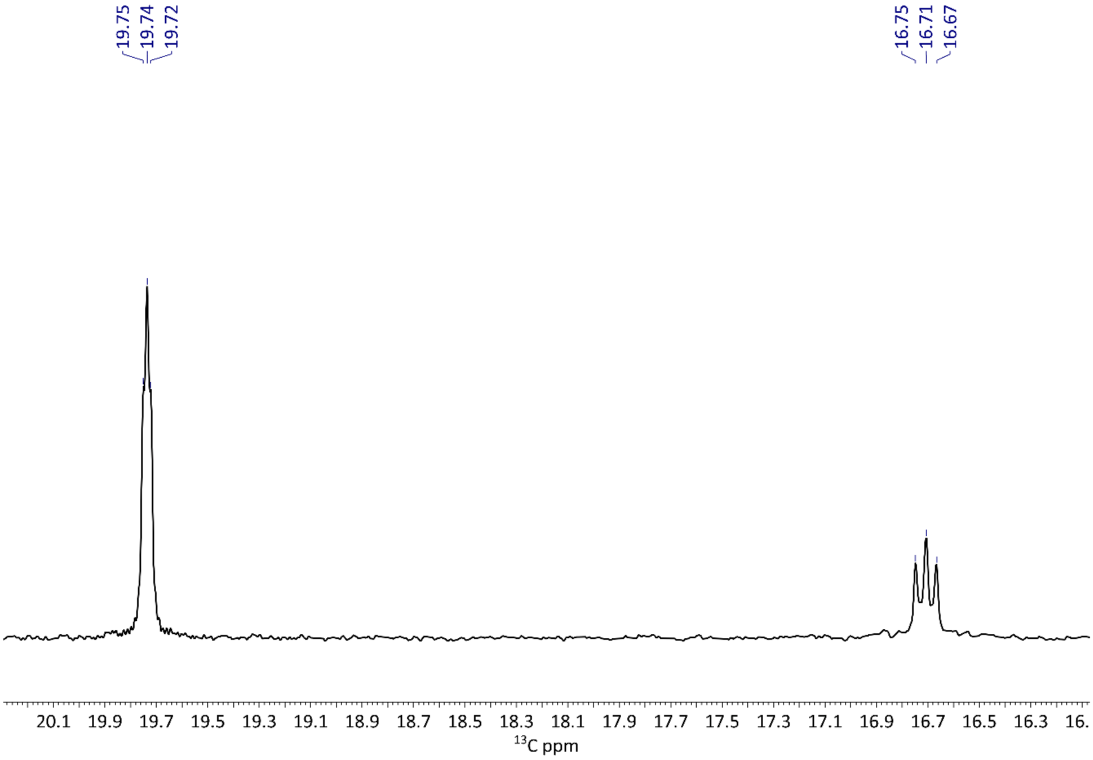

Figure S21 ^{13}C{^{1}H} NMR (126 MHz, 25 °C) spectrum for Hg[P(Si^{i}Pr$_3$)$_2$]$_2$ **4** in C_6D_6.

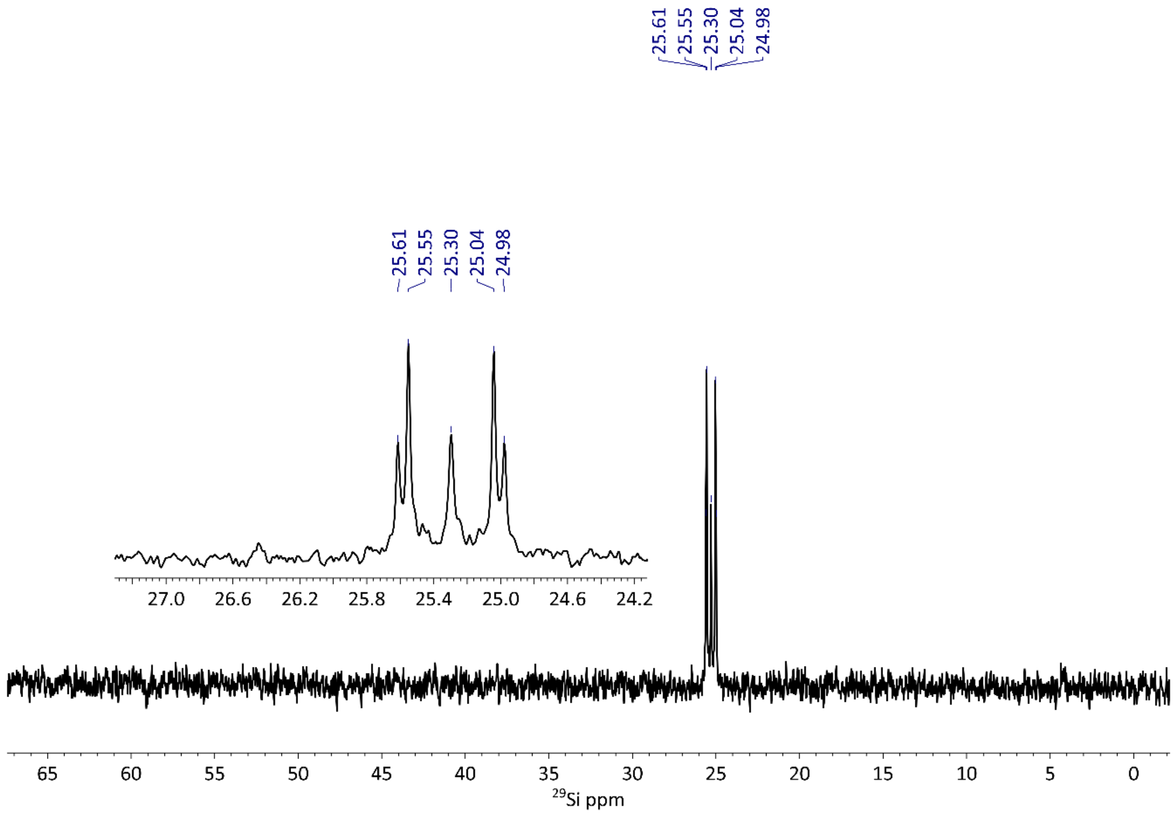

Figure S22 ^{29}Si NMR (99 MHz, 25 °C) spectrum for Hg[P(Si^{i}Pr$_3$)$_2$]$_2$ **4** in C_6D_6.

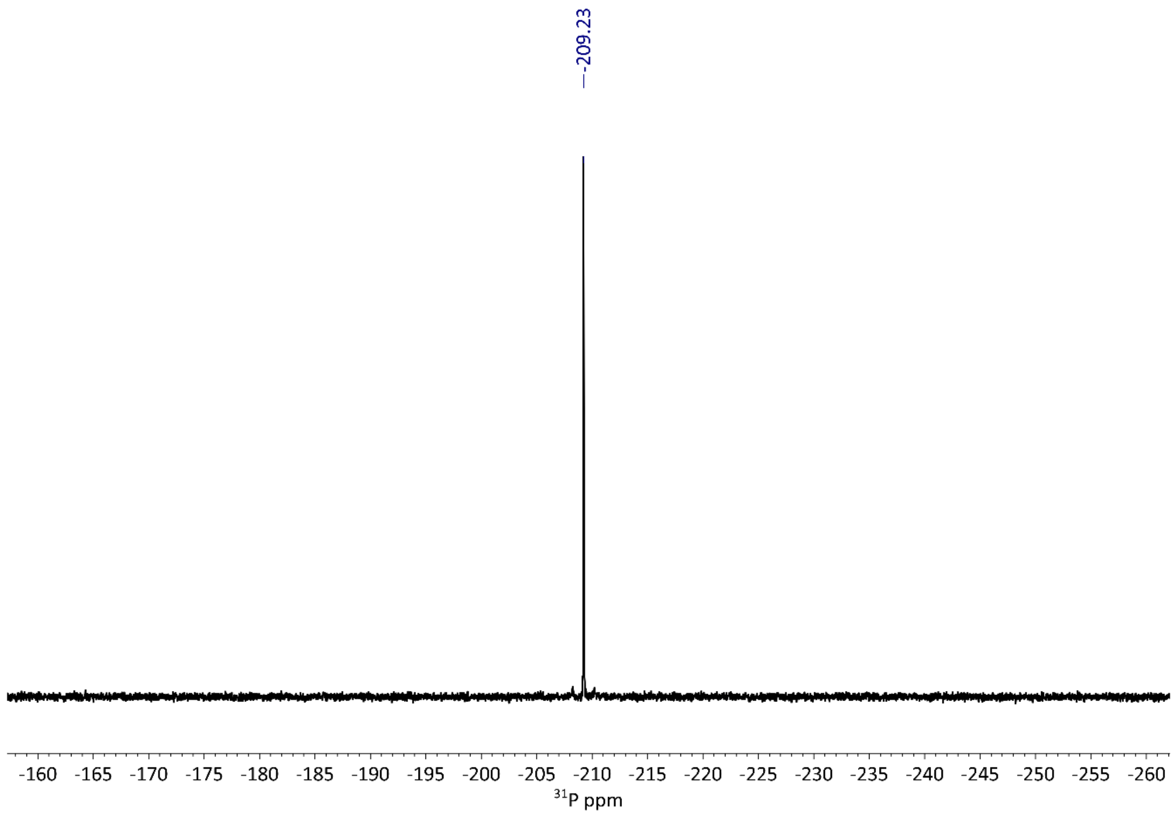

Figure S23 ^{31}P{^{1}H} NMR (202 MHz, 25 °C) spectrum for Hg[P(Si^{i}Pr$_3$)$_2$]$_2$ **4** in C_6D_6.

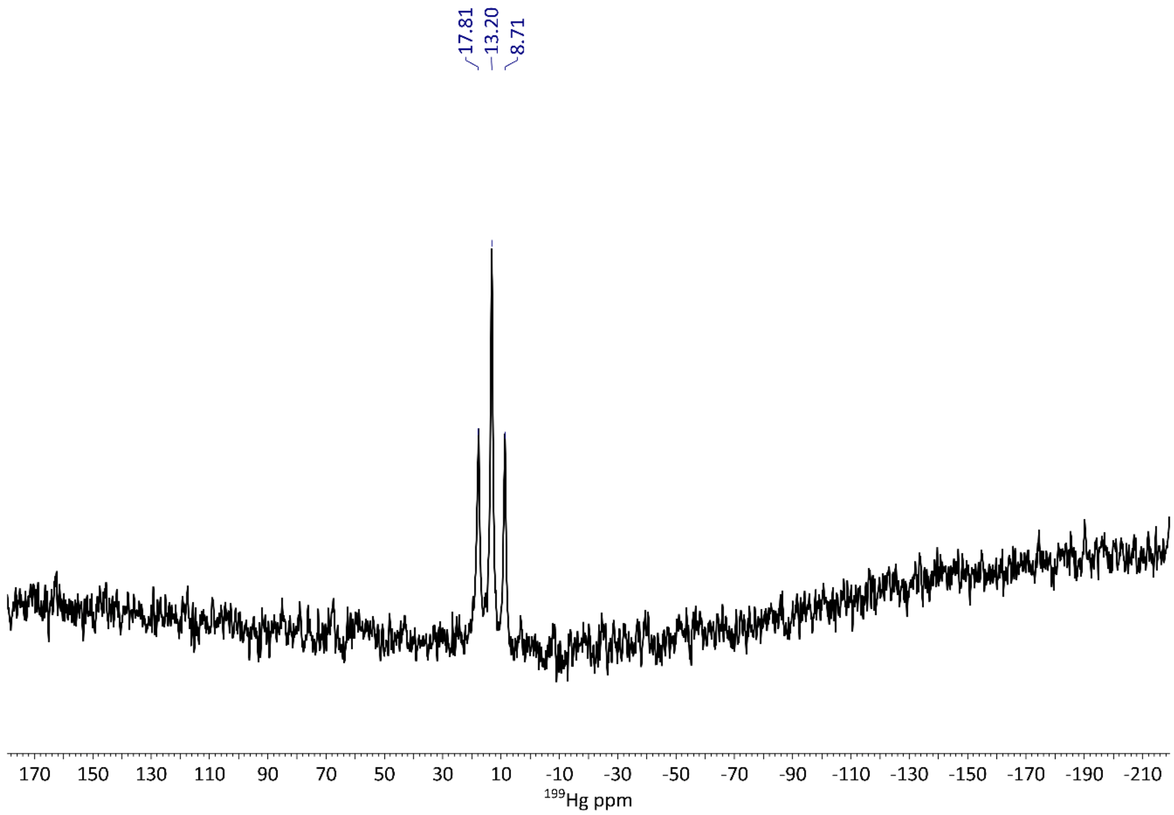

Figure S24 ^{199}Hg NMR (90 MHz, 25 °C) spectrum for Hg[P(Si^{i}Pr$_3$)$_2$]$_2$ **4** in C_6D_6.

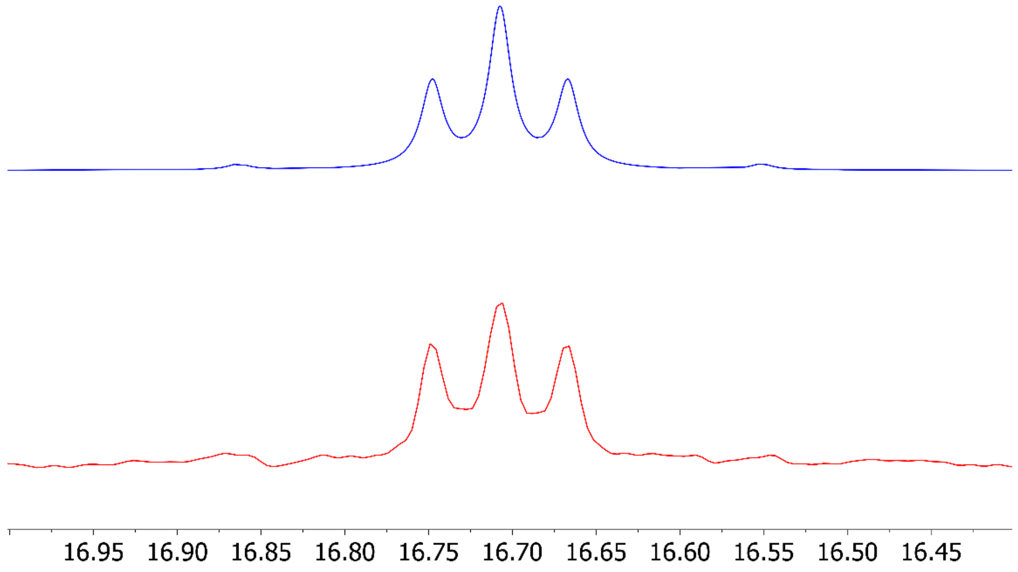

Figure S25 Experimental (red) and simulated (blue) $^{13}C\{^1H\}$ NMR signal for ^{i}Pr C̲H in **4**. $^2J_{CP}$ = 10.2 Hz, $^2J_{PP'}$ = 19.0 Hz.

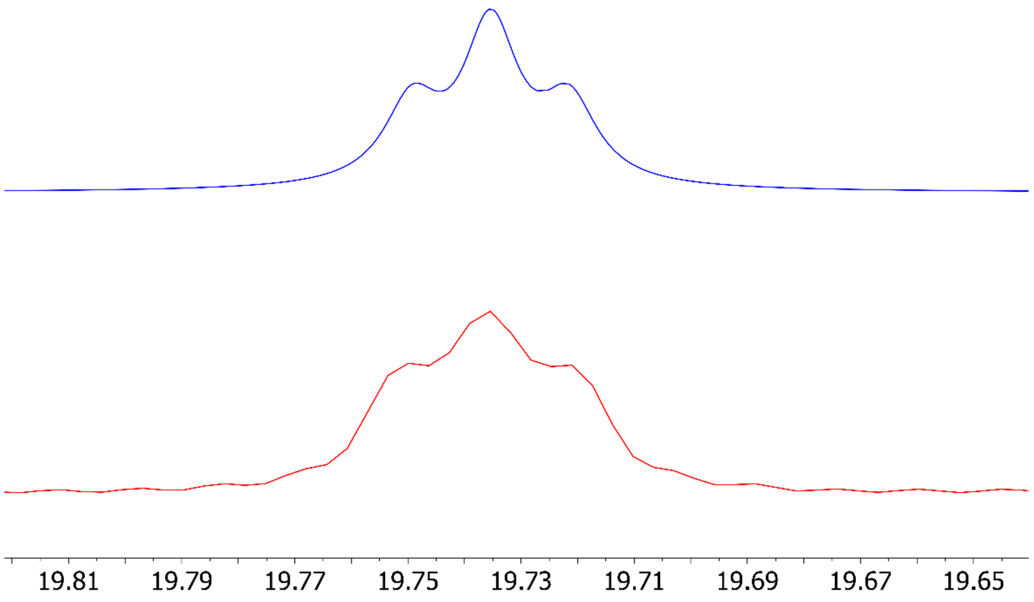

Figure S26 Experimental (red) and simulated (blue) $^{13}C\{^1H\}$ NMR signal for ^{i}Pr $\underline{C}H_3$ in **4**. $^2J_{CP}$ = 3.5 Hz, $^2J_{PP'}$ = 19.0 Hz.

S3 – IR Spectra

S3.1 – NaP(Si^{i}Pr$_3$)$_2$ (**1**)

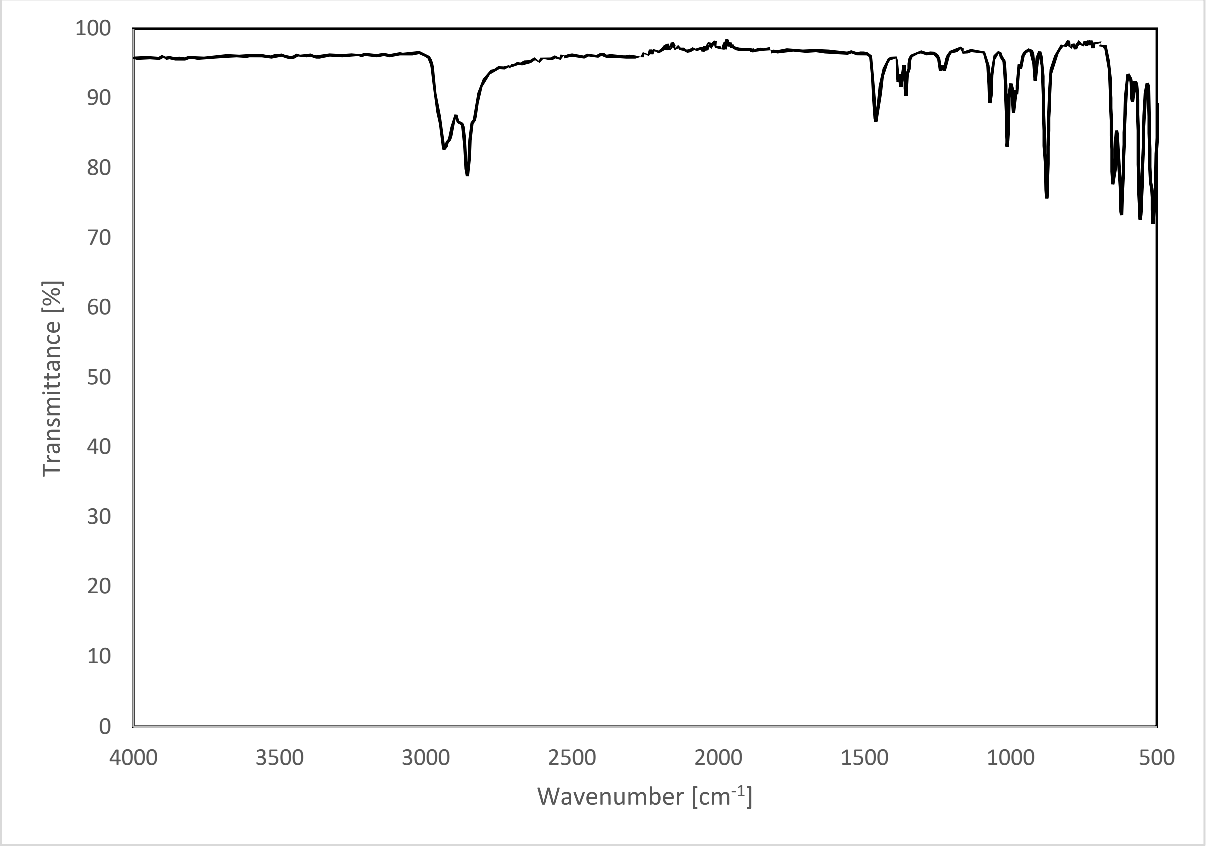

Figure S27 ATR-IR spectrum of NaP(Si^{i}Pr$_3$)$_2$ **1** (resolution 2 cm^{-1}, spectral average of 32 scans).

S3.2 – $Zn[P(Si^{i}Pr_3)_2]_2$ (**2**)

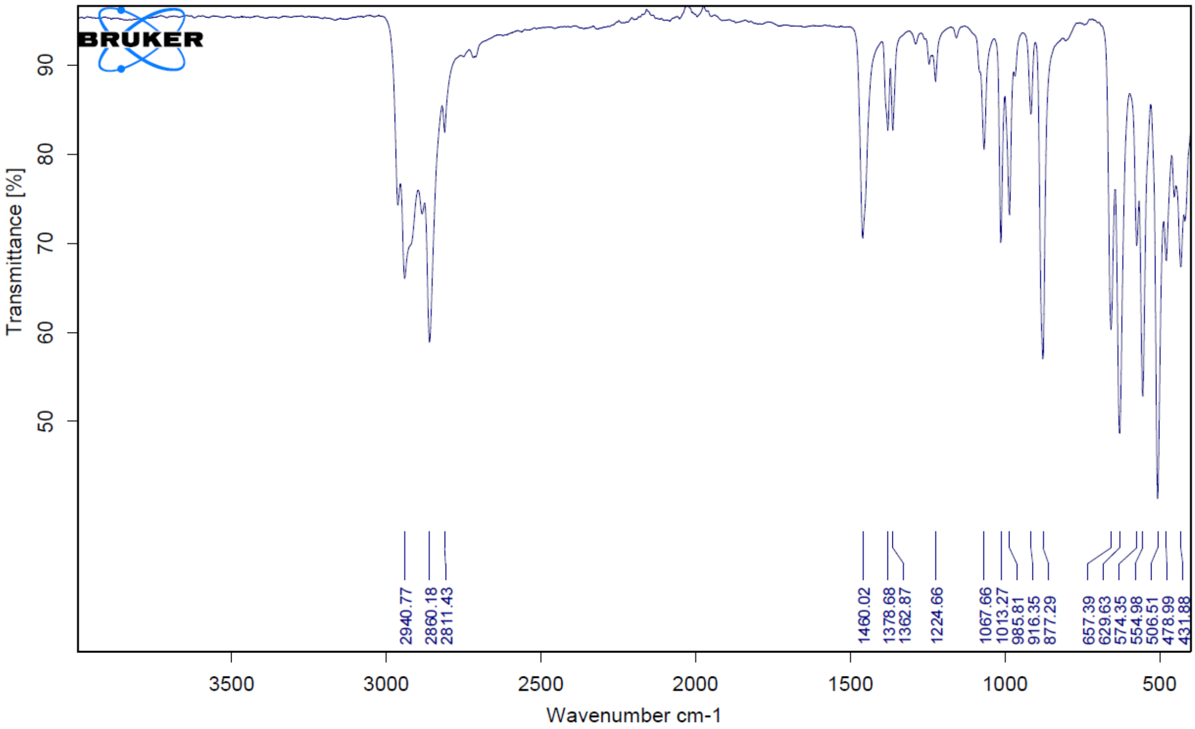

Figure S28 ATR-IR spectrum of $Zn[P(Si^{i}Pr_3)_2]_2$ **2** (resolution 2 cm^{-1}, spectral average of 32 scans).

S3.3 – $Cd[P(Si^{i}Pr_3)_2]_2$ (**3**)

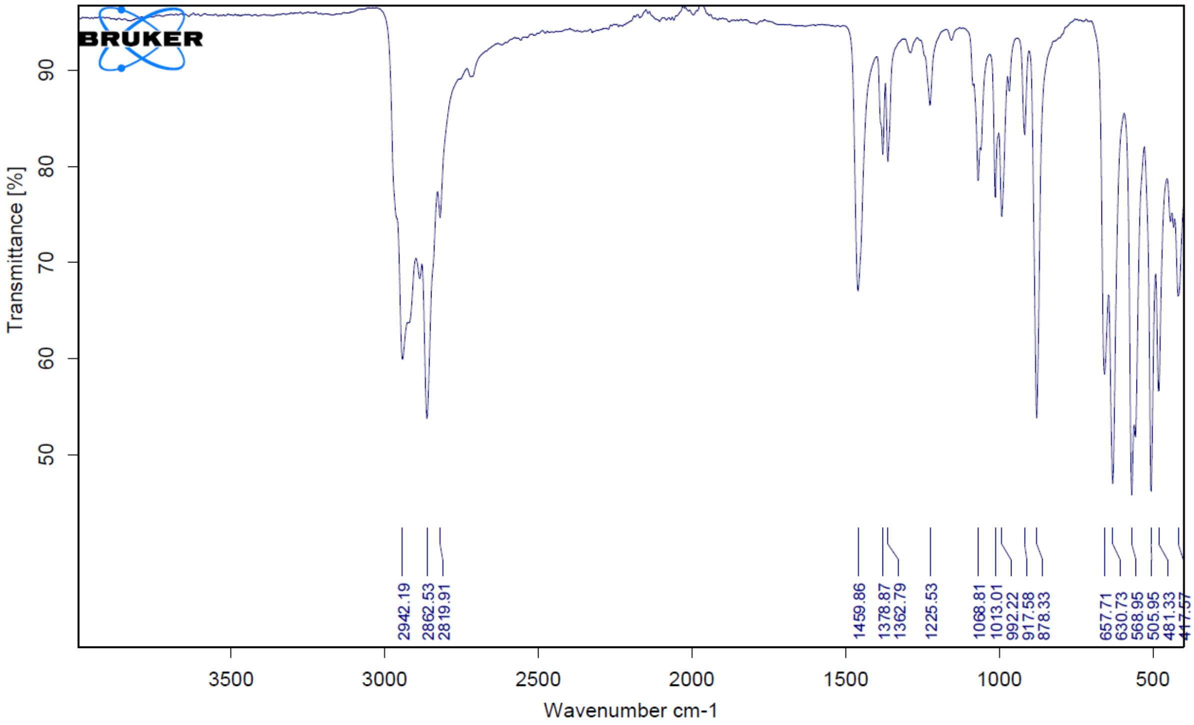

Figure S29 ATR-IR spectrum of $Cd[P(Si^{i}Pr_3)_2]_2$ **3** (resolution 2 cm^{-1}, spectral average of 32 scans).

S3.4 – Hg[P(Si^{i}Pr$_3$)$_2$]$_2$ (**4**)

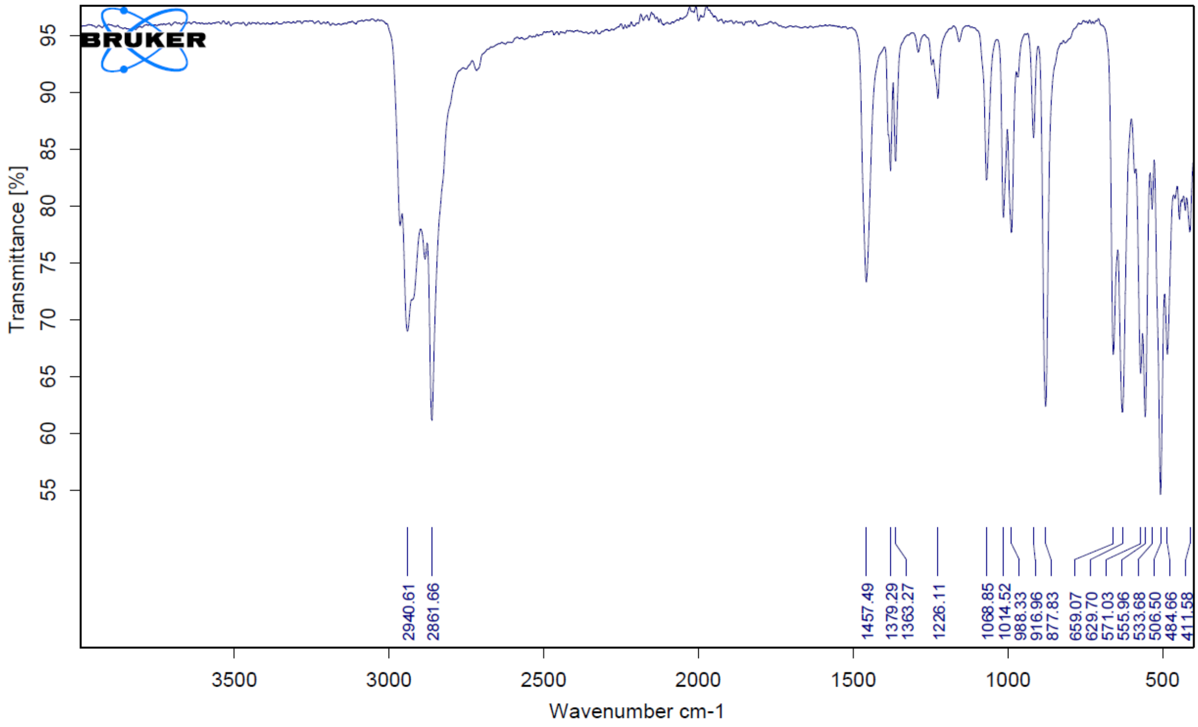

Figure S30 ATR-IR spectrum of Hg[P(Si^{i}Pr$_3$)$_2$]$_2$ **4** (resolution 2 cm^{-1}, spectral average of 32 scans).

S4 – Mass Spectrometry Data

S4.1 – Zn[P(Si^{i}Pr$_3$)$_2$]$_2$ (**2**)

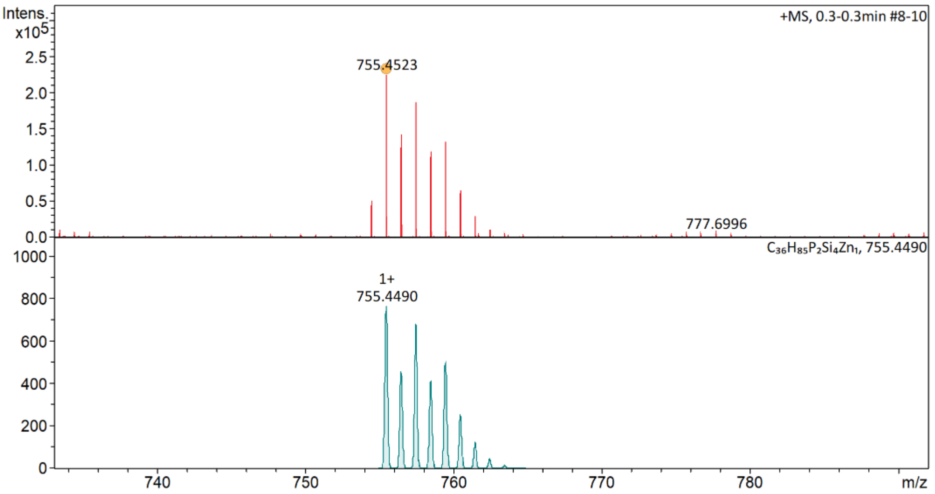

Meas. m/z	#	Ion Formula	Score	m/z	err [mDa]	err [ppm]	mSigma
755.4523	1	C36H85P2Si4Zn	100.00	755.4490	-3.4	-4.5	37.8

Note: Sigma fits < 0.05 indicates high probability of correct MF

Figure S31 HRMS of Zn[P(Si^{i}Pr$_3$)$_2$]$_2$ 2 showing the [M+H] peak.

S5 – Computational Methodology

S5.1 – Geometry Optimizations

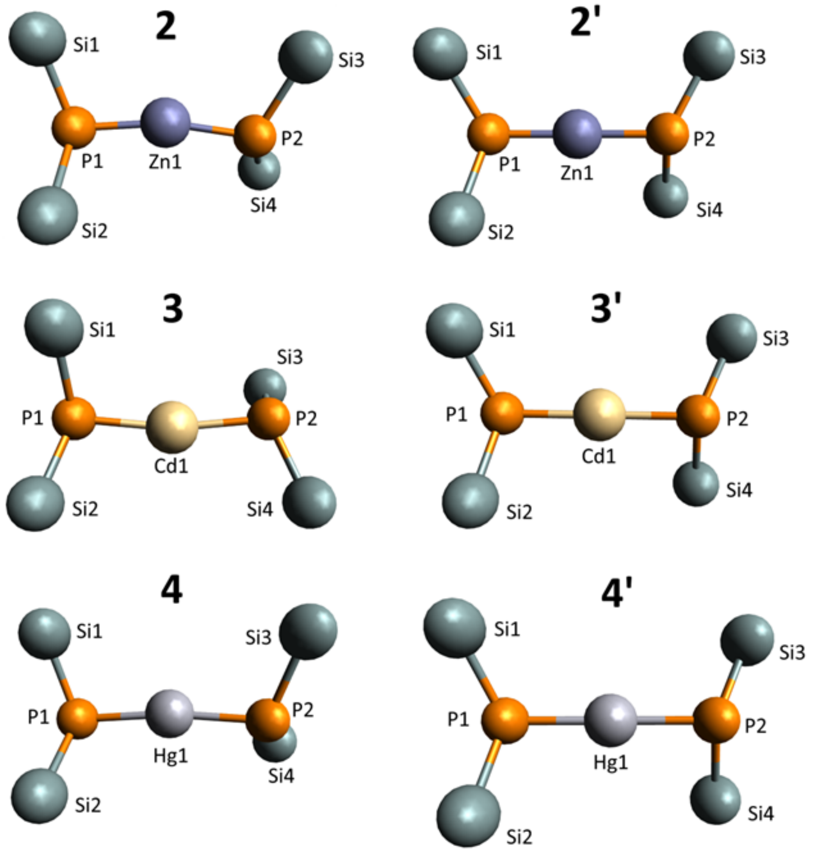

Figure S32: Optimized geometry of bent (**2**–**4**) and linear (**2'**–**4'**) metal phosphanide complexes, showing geometry around the metal and phosphorus centers. Carbon and hydrogen atoms are omitted for clarity.

Table S2: Selected bond angles (°) for bent (**2**–**4**) and linear (**2'**–**4'**) geometry optimized metal phosphanide complexes. Atom labels given in Figure S19. M = Zn, Cd, Hg.

Geometry	P1–M1–P2	M1–P1–Si1	M1–P1–Si2	M1–P2–Si3	M1–P2–Si4
**2**	168.1	104.0	99.0	110.6	106.0
**2'**	180.0	117.0	103.2	118.5	95.4
**3**	169.5	98.6	102.3	102.3	107.7
**3'**	180.0	114.3	102.2	114.1	95.9
**4**	172.6	102.8	102.3	106.8	102.9
**4'**	180.0	113.2	102.9	114.2	96.0

S5.2 – Geometry Optimized Coordinates

S5.2.1 – Coordinates for **2**

127

Zn	0.02239184715737	-0.11355480341434	0.05952172212749
P	0.11184807682847	-0.15364075993225	2.29317756216872
Si	-1.67797130333428	-1.37917981290414	2.88695653761888
C	-1.67319159048948	-2.78291736812753	1.59730354562636

H	-1.90655038176794	-2.25905174949060	0.65788391308725
P	0.23073315293984	0.27604952182383	-2.12189368910001
Si	0.28600783401741	2.05983221928470	2.67928129906029
C	-2.77586894200929	-3.81189218508746	1.83101928895185
H	-3.77191874858337	-3.36799478520528	1.80723029870651
H	-2.65748172413121	-4.31264354708739	2.79653232349777
H	-2.74372135244670	-4.58762570495595	1.05846961980797
Si	-1.42289019918450	-0.72546813437033	-3.26381221517633
C	-0.33426163352387	-3.48742686767316	1.40534241055445
H	0.48820344930697	-2.78935942496787	1.23359032341236
H	-0.38226790146226	-4.17182672626937	0.55268308584639
H	-0.07026001764127	-4.08349526439136	2.28066144298596
Si	2.39364390849273	-0.08732248377130	-2.61052188063780
C	-3.34531642325249	-0.46499757209200	2.84613331862855
H	-3.13276467230150	0.47147178609661	3.37485300872554
C	-4.47404338070010	-1.16251371120633	3.60334497227679
H	-4.75853034977337	-2.10560688767715	3.13296815384733
H	-5.36791342349117	-0.52993330456518	3.62031191311596
H	-4.20851730252262	-1.38193783419620	4.63898314142951
C	-3.78069413409427	-0.11739094021319	1.43019132131471
H	-2.98498834720725	0.37897397772337	0.86974562787185
H	-4.64765953617141	0.55159720074079	1.43183302411498
H	-4.06605839194618	-1.01487952812593	0.87349827570989
C	-1.42650178143452	-1.99757332124572	4.66390172561906
H	-2.27011594705455	-2.67847671384260	4.84438737518036
C	-1.53013679614667	-0.84753465477182	5.66202471520688
H	-0.68700870602095	-0.16235093328427	5.55050810874218
H	-1.50652534364019	-1.22362483174120	6.69009862207971
H	-2.44646521506635	-0.26466403866069	5.54501477918371
C	-0.13315553297860	-2.77169374468042	4.88795717244069
H	-0.11272688003407	-3.71114131108962	4.33428106987175
H	-0.00821388101003	-3.01364755937531	5.94887515605912
H	0.73613595969260	-2.18620018747539	4.57562721412833
C	-1.05415077176088	3.23962444157256	2.03079376210802
H	-0.59872459405378	4.22405317010840	2.21009623819538
C	-1.29056855552618	3.10503132564092	0.53443515482815
H	-0.37479374460932	3.20311990377999	-0.05295013377479
H	-1.99652237822233	3.86134562452374	0.17509567794628
H	-1.72438829420449	2.13027007361554	0.29579371779075
C	-2.37270510997846	3.20095219848844	2.79012852267793
H	-2.91520967215285	2.27827284210649	2.57994157480649
H	-3.02035750902403	4.02978988595249	2.48545773522749
H	-2.23878852462834	3.26886861735199	3.87215953987551
C	0.42231825565064	2.32659584754145	4.55462328369572
H	-0.55663629938832	1.98790359834162	4.92161411736296
C	1.48947166073083	1.48176353873641	5.24174871178338
H	1.45947373717639	0.43665271593308	4.92590786888960
H	1.36602619947222	1.51086269909581	6.32947604420452

H	2.48999735858142	1.85933275102444	5.02058270899025
C	0.57338045824610	3.79613524709201	4.94221394324843
H	1.54180490121689	4.19098380437469	4.62391985110010
H	0.51903264655525	3.91500795779158	6.02953350744376
H	-0.20105371844752	4.42899721599380	4.50381961676835
C	1.86385018670107	2.46382551942874	1.67719647342108
H	1.62372415501418	2.08127076781953	0.67236513554807
C	3.11620386456971	1.73481632280187	2.15085612095901
H	3.48195354243748	2.15553320654843	3.09050532097225
H	3.92384350050806	1.84060644663269	1.41805026392931
H	2.93962149982799	0.66934868802808	2.30882986426872
C	2.14703085595844	3.95556033715159	1.52436361688025
H	1.30814842012384	4.50098292189894	1.08868947114547
H	3.01311511965983	4.11238823576512	0.87251353056064
H	2.38087459658485	4.41806774714497	2.48682888366237
C	-2.95699541535739	-0.27918473473497	-2.22544002620532
H	-2.67245299925154	-0.62194121931948	-1.22018726518891
C	-4.22281812763812	-1.03645509035352	-2.61616380183065
H	-4.56219876216965	-0.75704406114031	-3.61701276181793
H	-5.03730588271198	-0.79877972811061	-1.92323881305257
H	-4.08812584993630	-2.11972111524185	-2.60401660524001
C	-3.24290766725791	1.21577447650126	-2.13582382164980
H	-2.34116633337340	1.80089520538268	-1.94816909696330
H	-3.95721728099396	1.42688746853805	-1.33319443599783
H	-3.68670882137942	1.58064337037972	-3.06519113205198
C	-1.38271240137445	0.01069009344696	-5.01555589555617
H	-0.40329720442643	-0.30839819652209	-5.39719239873235
C	-2.45165184070738	-0.58428877586830	-5.92944608483912
H	-2.41735049986777	-1.67531413598150	-5.96303355046074
H	-2.32970841044724	-0.21944656291538	-6.95482744657302
H	-3.45458072689068	-0.29519831567910	-5.60381806612821
C	-1.42174306674949	1.53410283344898	-5.07147226414595
H	-2.42329738986558	1.90913344353823	-4.85086414078128
H	-1.15625518250586	1.89124244038789	-6.07193779142742
H	-0.73745862542361	1.99152903184278	-4.35361699360736
C	-1.40778951320553	-2.61517197919695	-3.43696992343567
H	-2.36488240934445	-2.83061718355409	-3.93280305585616
C	-1.41253615954220	-3.31865293110153	-2.08816756903327
H	-0.53673828410681	-3.03975761462688	-1.49756623268415
H	-1.39306262075351	-4.40704347592468	-2.20938775185466
H	-2.29643810782518	-3.07045259044345	-1.49617811015583
C	-0.29575250941083	-3.14347472400285	-4.33251279226491
H	-0.31742105370977	-2.69610213827051	-5.32891279100170
H	-0.37703992203119	-4.22859707870091	-4.45626644255791
H	0.68457654796814	-2.94142440845548	-3.89930629086897
C	2.54602133243445	-0.41058799600947	-4.47850252496097
H	1.75134662872900	-1.13648160483538	-4.69003580266485
C	3.87139683331420	-1.05072780383779	-4.88373614559112

H	4.71844906183127	-0.39348185956718	-4.67048695010833
H	3.88350062116750	-1.25301777656550	-5.96010418791406
H	4.05253217973524	-1.99723770167958	-4.37095717439484
C	2.27733293738270	0.83340851739266	-5.31954987536425
H	1.36755469975530	1.35378479568023	-5.01721529165367
H	2.17751004891060	0.57328609738671	-6.37875082743210
H	3.10497325450731	1.54313347077036	-5.23968363779030
C	3.24362626449259	1.51137156369580	-2.01403673986769
H	3.11523179969020	1.45799494654090	-0.92400436158148
C	4.74439280501504	1.50937541568200	-2.29640592920715
H	5.23888766012746	0.60538365418171	-1.93265386785809
H	5.23102882143740	2.36554820528470	-1.81716685870473
H	4.94388469613011	1.58696119624904	-3.36891682628314
C	2.60899874758892	2.82008916549864	-2.46886597142984
H	2.75453936596403	2.98775081224975	-3.53792679716955
H	3.06131064120619	3.66744112078322	-1.94240950395641
H	1.53444044758382	2.83499113984634	-2.27617778796971
C	3.24266707270344	-1.50972681758305	-1.66874474665093
H	4.25454008520059	-1.52724330165051	-2.09776274303399
C	2.61194343046049	-2.87180722640268	-1.91546100124886
H	1.58268306582869	-2.89566546530684	-1.54997534668770
H	3.16468732032004	-3.65778206373650	-1.39014970388364
H	2.59198751809772	-3.13725359806431	-2.97448802026014
C	3.38453860770169	-1.25493946129170	-0.17451894766754
H	3.95242824305418	-0.35004690192404	0.04576299200107
H	3.89503974989573	-2.09172550195152	0.31392159777734
H	2.41200640398994	-1.15797899022143	0.32136192491939

S5.2.2 – Coordinates for **3**

127

Cd	0.12373756485539	-0.07635866652933	0.05217512137673
P	0.13736268137476	-0.23397814968482	2.46546449986574
P	-0.27835906501209	0.28093895410080	-2.29794753656034
Si	-2.25522450289762	-0.74160706256090	-2.60561295920433
Si	2.28974732808061	-0.71597788449755	2.89997362138086
Si	-0.70083176451752	1.80427308891002	2.93930045668758
Si	1.54051831117054	-0.42566835734168	-3.42024289826635
C	2.60656268923430	-2.28087776527481	1.85515529240229
H	2.65582051099130	-1.89604144261461	0.82672628841535
C	-2.57552576767406	-0.84432311623424	-4.47648398179911
H	-1.83654878803351	-1.57936639900987	-4.81995318832967
C	-1.85709575815308	-3.62337668752543	-2.55569105902677
H	-2.07562643710157	-3.65619838479795	-3.62523813555815
H	-2.17170215649177	-4.58123954550166	-2.12759387055625
H	-0.77331947758766	-3.55954004261747	-2.43570282597348
C	-2.55331476492812	-2.46702844103173	-1.85390497960188

H	-3.63472580695651	-2.59526858238153	-2.00275907800949
C	3.55555992607716	0.61858641223749	2.38695917117359
H	3.07992404426263	1.56769011113734	2.65376375601558
C	-3.42819773091135	0.42912415927774	-1.65669670182380
H	-2.95853711023672	0.49195354257625	-0.66521476026736
C	-1.14047565155841	1.86722863884453	4.78917336051003
H	-0.17321101380365	2.04527786488570	5.27586861439997
C	-1.71052395832379	0.57575521604330	5.36312186715430
H	-2.69677701326756	0.35531180456680	4.94939195202162
H	-1.82086090222427	0.65256035467041	6.45013939959129
H	-1.07361700111007	-0.28379745022692	5.14646160911106
C	2.23620511681501	0.20505243833439	5.57975642193103
H	2.80133084420980	1.07311439644860	5.23222059839698
H	2.49054333348867	0.04361567107593	6.63253171319723
H	1.17716687010416	0.46661266483691	5.53949750308524
C	-2.29218194045036	-2.52731521832772	-0.35481334808175
H	-1.21772840170602	-2.50697201022924	-0.14330623366793
H	-2.67040099799095	-3.46063800138299	0.07510404670611
H	-2.75370763820515	-1.70389478272054	0.19555942850421
C	-4.83859130847978	-0.11444540677713	-1.44544451414195
H	-5.36989631991418	-0.23963325026016	-2.39217495320940
H	-5.42765949780612	0.58134433893474	-0.83777702026965
H	-4.84563138816197	-1.07815611319799	-0.93283405645458
C	-3.95935151323621	-1.39452790813291	-4.81348666950653
H	-4.16849651173624	-2.34366630288003	-4.31433123489290
H	-4.05623679394856	-1.56203448816953	-5.89146502783414
H	-4.74454081423272	-0.69021493670385	-4.52714052106452
C	2.00022602969987	-2.80638419574763	-1.82123500732605
H	1.04601541059931	-2.60545299457877	-1.32690814696486
H	2.65013954602381	-3.30875184473880	-1.09747025591437
H	1.80418289350360	-3.51563269328459	-2.62766756379121
C	1.50230536102417	-3.33138319530371	1.88833166700605
H	1.42726449892906	-3.80494927697754	2.86907664341018
H	1.70495830612640	-4.12313826813130	1.15905920974618
H	0.52167278339145	-2.90739920918372	1.66390212714006
C	3.95751171527237	-2.92094496261555	2.16525242213763
H	4.79053939962582	-2.22844434471185	2.03059875144256
H	4.13615532997194	-3.77901294817871	1.50806900496290
H	3.99706182286191	-3.28989927403789	3.19430485932917
C	2.51022837214692	-1.04710231405301	4.75452606390664
H	3.57378317241794	-1.30002872866877	4.86374259488219
C	2.65020665048228	-1.53083145062816	-2.33798599849089
H	2.85216970837627	-0.89190139513481	-1.46637477554901
C	3.79409776388341	0.64018041254671	0.88482498267704
H	4.32008504359961	-0.25720523694121	0.54678181245183
H	4.39850111896522	1.50436651393077	0.59131673620481
H	2.85965031352741	0.70163468299726	0.31915298498044
C	-3.28883719949332	0.81412934324505	2.16636030239018

H	-4.06980928669011	0.76283766494629	1.40048169100891
H	-3.78141281193876	1.06627054662590	3.10892146786832
H	-2.85496747682934	-0.18237953882415	2.27681194861441
C	3.99065006400488	-1.83693196850346	-3.00032746920180
H	4.65945270719147	-2.35364372057613	-2.30359260443373
H	4.50249720496018	-0.93377898993811	-3.34054415081155
H	3.86068146298576	-2.49175861932878	-3.86610119743331
C	4.88600755114630	0.55325982404597	3.13382005282613
H	4.76030837859374	0.62186389348723	4.21555742986024
H	5.53623094597992	1.37954316788265	2.82702857919379
H	5.42670209546184	-0.37354193662319	2.92652275445056
C	-2.04160892291689	3.05274853786898	5.12914105125571
H	-1.66321891784803	3.99811307636331	4.73158921757109
H	-2.13742652388479	3.16682598933607	6.21406991786647
H	-3.04932615700894	2.90704784797660	4.73185790312943
C	1.69025488121638	-2.22022118110275	5.27749751016212
H	1.79412477622545	-2.31007507042784	6.36421780185888
H	2.00647223076012	-3.16811572871831	4.83911648273955
H	0.62665360721375	-2.09363247901802	5.05706879983586
C	2.97492676801243	1.86796536421866	-2.46958592492194
H	3.67382720563097	1.23525379059549	-1.91938585992309
H	2.14354495836420	2.11296384941327	-1.80068694832821
H	3.49120406901430	2.80690595926431	-2.69666799652441
C	2.47309587696124	1.20204180933919	-3.74381912900132
H	3.34763331525227	0.92359638918150	-4.34683080739600
C	-2.24058275120148	1.86021454212547	1.80273744206639
H	-1.84815448420193	1.59872057783476	0.80748138080706
C	-2.31639206256775	0.45431676898152	-5.23352425574454
H	-3.09329959339182	1.19324740291307	-5.02720659560653
H	-2.31434634357858	0.27890040524367	-6.31464939721468
H	-1.36148224902448	0.90867929290626	-4.96153077715223
C	-3.48163701512831	1.84632276479847	-2.21585151793278
H	-2.48455014625863	2.24887462989723	-2.40539958108216
H	-3.99253399890374	2.51793523658259	-1.51786310954803
H	-4.04008777518776	1.87632360631139	-3.15482730196638
C	1.12757377096651	-1.17599787102128	-5.12354068523694
H	0.25465446798790	-0.60871987916010	-5.46696261470835
C	2.24239336201204	-0.96818644868830	-6.14821756237228
H	2.48319934479165	0.08406342193688	-6.30357449658980
H	1.94415382311577	-1.38335166679433	-7.11700312626727
H	3.16323998302248	-1.47638919134826	-5.85061408995635
C	0.74037512004476	-2.64894420137949	-5.08243421423390
H	1.60985685808641	-3.27616128694963	-4.86824414836409
H	0.34375386835619	-2.97214788926217	-6.05090976680527
H	-0.01361408148481	-2.86427098856592	-4.32665546758822
C	-2.88138632211770	3.23681330537677	1.65234958780239
H	-2.18296120358567	3.97755875721117	1.25897156563033
H	-3.26731142928942	3.61338748079893	2.60205922461172

H	-3.72534933255982	3.18485258044864	0.95642471843281
C	0.32756683760141	3.35250505260083	2.53396861254789
H	-0.41820592753279	4.15870556896065	2.55065594302198
C	0.91430734851834	3.30685007681746	1.12866662208106
H	1.38560353936552	4.25928767469164	0.86441877958138
H	0.15510460479658	3.09911177953143	0.36804496942878
H	1.68089626979967	2.53218506793848	1.04866712627133
C	1.38132401788514	3.70064195132265	3.57778664175725
H	2.09537929922055	2.88847002642260	3.72916384475868
H	0.93361101393380	3.92041449741943	4.54925285556252
H	1.95399302752948	4.58294559351496	3.27288608100086
C	1.62657840045056	2.17963227569186	-4.55316928240859
H	1.25816809125691	1.74594469988297	-5.48616735759721
H	2.20259473029644	3.07488136838994	-4.81002938757199
H	0.75511947017597	2.49842265906088	-3.97508876213889

S5.2.3 – Coordinates for **4**

127

Hg	0.11942425975055	0.01903203162442	-0.04206154131450
P	0.06388061301139	0.04510849069813	2.35550926556112
Si	-2.14636430495210	-0.09880694085457	2.80486429641233
C	-3.18787147196060	1.43443560665406	2.34875365673906
H	-2.56081342387127	2.28367436093745	2.63988176956093
P	0.48496534481447	-0.01414411831136	-2.41152848219741
Si	1.18444477211530	1.94717487713006	2.84163033066493
C	-4.50390296609248	1.55860685569031	3.11386706918229
H	-5.18732503048132	0.73787408843447	2.88422766567433
H	-5.01537205943875	2.48791669016331	2.84122551657636
H	-4.35911618129406	1.57010979420454	4.19521135005066
Si	-1.56124184916868	-0.03066006012035	-3.38939781174421
C	-3.43358237982213	1.54503414081490	0.85063473493717
H	-2.50973358518716	1.44329125347983	0.27596403490515
H	-3.87018781131013	2.51500448328063	0.59134111723372
H	-4.12364114698337	0.77297838822654	0.50024885331687
Si	1.83674694388132	-1.80050163586457	-2.68955718179961
C	-2.71673670102659	-1.57340316862049	1.74147711367660
H	-2.65468279822948	-1.18328204855641	0.71654995234907
C	-4.17458854639662	-1.94401698516492	2.00186194877685
H	-4.32196291707916	-2.28332019570450	3.03144550434166
H	-4.48664964457280	-2.76411263606474	1.34589011953898
H	-4.85638888084625	-1.10986635187378	1.82796795834791
C	-1.83839892820998	-2.81828163743251	1.80147269616793
H	-0.77872982162904	-2.58723788273862	1.68237892812578
H	-2.12348510242815	-3.52247271028482	1.01283125953663
H	-1.94922730993241	-3.33918575493483	2.75427623402704
C	-2.39445891085267	-0.43086561656807	4.65648547364039

H	-3.48411061767261	-0.53273887074678	4.75691473857202
C	-1.95120624900710	0.74710961157034	5.51676753382665
H	-0.86346998313493	0.82552792249470	5.52147117361396
H	-2.26916696119278	0.61187761553110	6.55582678330460
H	-2.34907463484523	1.70433931577446	5.17115350946717
C	-1.75317608306523	-1.71902847601298	5.15840476366610
H	-2.20274583695571	-2.60449012419463	4.70763358672641
H	-1.86901005498928	-1.80811413023401	6.24396918762858
H	-0.68253595718880	-1.74432818797767	4.93745692335141
C	0.41930984172242	3.62028964725050	2.34119742990279
H	1.29479955275301	4.28124804082782	2.25993423914013
C	-0.50563023059844	4.21221077140002	3.40025196173635
H	-1.33215291025957	3.53794444396914	3.63874779355634
H	-0.94626679635084	5.14995521800136	3.04577216976843
H	0.01507638278903	4.42637929446005	4.33487262026479
C	-0.27019309925559	3.59855888716694	0.98272943873361
H	0.37019928270845	3.20480409723366	0.19145612311530
H	-0.57871418378551	4.60640768887864	0.68488744899573
H	-1.16725510209039	2.97907799056276	1.01253743487999
C	1.48157601012547	2.00727270985350	4.72219891011493
H	0.50773367723274	2.29698303518913	5.13416463536914
C	2.47752416058602	3.10445013374212	5.09495010436772
H	3.49062161718223	2.84206431787422	4.77860146845921
H	2.50778933290320	3.24649268575826	6.18033903867718
H	2.23436237622906	4.07050576741326	4.64514119591239
C	-2.93136653179631	2.15929814936817	-4.74514686226774
H	-2.85692270583629	1.63195414482580	-5.69755253869475
H	-2.93114682012693	3.23208090907987	-4.96711939671427
H	-3.90610172124530	1.92167031143769	-4.31502698060591
C	1.88451313315446	0.68553440589135	5.36527409642193
H	1.23224359838465	-0.13704341449892	5.06604211306743
H	1.85871518620137	0.76048840156596	6.45764980497505
H	2.90384048475864	0.40585729853674	5.08797747875260
C	2.88264045301018	1.80794630986333	1.97889194349354
H	3.50176264811363	2.47451453608786	2.59584288542135
C	2.94157172200311	2.35103541023319	0.55692053879109
H	2.34098902173038	1.75647401846159	-0.13866358229681
H	3.96896654175878	2.32318910369018	0.18023923531882
H	2.59532776003725	3.38386314225739	0.49011603601720
C	3.50100419474596	0.41608223542556	2.04516827323359
H	3.54255447379252	0.02312519428697	3.06176884112968
H	4.52087985771480	0.42664928895089	1.64607630961620
H	2.92459828635401	-0.30030439285015	1.45492533883120
C	-1.78427682773038	1.81601293809813	-3.79851641901090
H	-0.84570358781827	2.06356216799653	-4.31218086500498
C	-1.84683403612726	2.66990157629230	-2.53654050499864
H	-1.84904837503365	3.73622296462598	-2.78569074823250
H	-0.98961574454128	2.48899979089919	-1.88371910041468

H	-2.75491329822510	2.47191418614130	-1.96033194682179
C	-2.89788122526054	-0.63153440322426	-2.17280831969803
H	-2.62239628474300	-0.12651250048130	-1.23873451674427
C	-2.83229206190489	-2.12914161589262	-1.90901041304514
H	-3.06161755246113	-2.71095965619031	-2.80545367637746
H	-3.55718542028248	-2.42104293571338	-1.14190059400757
H	-1.84369162764728	-2.43146407646264	-1.56268007611877
C	-4.32343896561832	-0.20063798084850	-2.50497560570275
H	-4.43147376621675	0.88445394520281	-2.51956099660793
H	-5.01778686645168	-0.58723940372749	-1.75052237554628
H	-4.65594931658112	-0.58112278511906	-3.47355913110342
C	-1.48182980110345	-1.06105515761910	-4.98429045398224
H	-0.96502220016944	-1.98285007684659	-4.69332345386301
C	-2.84186573573232	-1.45666178100120	-5.55475493039913
H	-3.44106870277923	-0.58239206529729	-5.82053779174099
H	-3.42755498460653	-2.05736733300156	-4.85723363188921
H	-2.71240958477203	-2.04870132427645	-6.46720655721429
C	-0.63714234655132	-0.36849709133041	-6.05077981274728
H	-0.44238041294386	-1.04369995093155	-6.89071751544473
H	0.32588562317275	-0.03226202490211	-5.66526472036572
H	-1.15005130224488	0.50802680132738	-6.45381019461983
C	2.32304084683316	-1.84514435648981	-4.52782545656945
H	1.36822296972187	-2.03254795799141	-5.03627844111631
C	2.88486040304669	-0.53723011651626	-5.07700488714146
H	3.91679852176012	-0.38836606147807	-4.75361669135600
H	2.31477542146032	0.33371204899812	-4.74604829122479
H	2.88731080254982	-0.54705149143797	-6.17189038627468
C	3.25552078988260	-3.00635390485511	-4.86717041079541
H	3.42432972578476	-3.06114018210847	-5.94798322472762
H	2.85828472003406	-3.97236644803025	-4.54835575689058
H	4.23391250446272	-2.87966202782157	-4.39646337920319
C	3.27719354945895	-1.40885149476973	-1.50138403852343
H	2.77059213039332	-1.20183731147741	-0.54795618214919
C	4.06991508585329	-0.16197727968792	-1.87686410386415
H	4.69703754727853	-0.35098039084202	-2.75095868049053
H	4.73544606669614	0.13150100375279	-1.05912346834802
H	3.42381000863793	0.68694019998408	-2.10752229981055
C	4.22354951630298	-2.58147970771654	-1.25976934400746
H	3.70712051108966	-3.47169673992042	-0.89676519405646
H	4.97787387397451	-2.31175769460525	-0.51299958563286
H	4.75773576262687	-2.85858465953499	-2.17222786501459
C	1.21128208166024	-3.53216426303538	-2.22221149929613
H	2.11434206712948	-4.14921316755753	-2.33225126275597
C	0.76505204113498	-3.62759606995449	-0.77023610943559
H	1.54311538436929	-3.31069975564306	-0.07145282256463
H	0.48546274296993	-4.65374021062592	-0.50910834763083
H	-0.10941195539086	-3.00118729700615	-0.58297537036223
C	0.15741058345866	-4.09845549899235	-3.16444247420441

H -0.76545243833767 -3.51822531453432 -3.12497982988176
H -0.09760862876731 -5.12752593229321 -2.88927897378015
H 0.49585848989622 -4.11098790217099 -4.20304685111929

S5.2.4 – Coordinates for **2'**
127

P 8.88760615478684 3.17697573407442 -0.61692605937534
Si 10.17518947683261 1.96783961863316 0.76589946123857
Si 9.24238915395050 5.32986833725336 -1.20820937087548
C 11.90380821928079 1.76956027367239 -0.02574866963711
C 9.36315133719601 0.23822730794224 0.73684205793465
C 10.38799113045110 2.41812922765931 2.60773313173468
C 11.07329451009702 5.83369865249261 -1.25598407264325
C 8.49760493962720 5.24095371479242 -2.96447645272171
C 8.16216152970650 6.50969066310105 -0.16167053598881
Zn 6.72616622466064 3.45624248613368 -0.09559898270094
P 4.57110151290782 3.73468555974235 0.42419047644190
Si 3.62841167771544 4.22550194986357 -1.54913517297012
Si 3.65414002914762 2.22987065023802 1.82146536830756
C 1.77637401481026 4.54815921373298 -1.27465226683906
C 4.62673867085277 5.75689343918647 -2.10020677416416
C 3.81779202986403 2.94970479381306 -2.94875985131220
C 1.75582360968001 2.24772137003795 1.66118121909149
C 4.10317028709896 2.92988668142327 3.53156412142028
C 4.41117042124209 0.49597543432700 1.68977890938058
H 3.47457881520945 3.50016404799597 -3.83588415146250
C 2.94408872441960 1.71166024606825 -2.81092370995203
C 5.26978735879083 2.55662101226545 -3.18401705805391
C 4.38305984888304 6.15765067250302 -3.55299663291392
H 5.67106704652612 5.41715920738690 -2.03115229754822
C 4.48625496984944 6.96511647324411 -1.18193428971518
C 1.03755967569408 4.91268699048509 -2.56062536339884
C 1.45703788099837 5.55111606611202 -0.17022426786860
H 1.40078561662989 3.57054211448294 -0.94955690055039
C 4.40818709472271 -0.11516231203720 0.29650727843489
C 3.82886214397405 -0.47206272175497 2.71729685883952
H 5.45837254080778 0.66884866822246 1.96084316407686
C 1.21965182825583 1.33768420452493 0.56166962602407
H 1.50681291418682 3.28210265666381 1.39910526899864
C 1.05262976593504 1.92450181226769 2.97949733765341
C 5.60056946691072 2.88336890601236 3.80192502906358
H 3.60371696127705 2.26895269131342 4.25285193113906
C 3.57818822108469 4.34782494640784 3.73411528779103
C 9.70553850504933 -0.61558075294017 -0.47836755713804
C 7.85227373979012 0.27662508160663 0.90935038567786
H 9.79609620852557 -0.24779861652822 1.62434715424827
C 10.83202712100001 3.84383831848507 2.90066511689479

H	11.20083166914723	1.74032466558746	2.91382327785209
C	9.16773212413702	2.09011478259704	3.45834242228961
C	11.89317111732227	1.81490157570172	-1.54980525158618
H	12.15605640326782	0.74092537168015	0.27450457511888
C	13.01007339187377	2.66730868347075	0.51483678557461
C	8.40733523695424	7.99297128196813	-0.43885578481258
C	8.18979809083263	6.26638935916214	1.34183632337662
H	7.14372687352152	6.27797085126065	-0.51152150916006
H	11.59679025785030	4.93772468216635	-1.61175803302030
C	11.61945348019170	6.18142476584549	0.12315134304446
C	11.37602125449821	6.96374783766549	-2.23820885528447
C	9.36304397033110	4.41159772593827	-3.90791374046089
C	8.07833033873665	6.55932555000131	-3.60444782044979
H	7.58009138513414	4.65858383273934	-2.79761720545781
H	11.13026281120851	1.17107125997024	-1.98718196456041
H	12.86670589406726	1.51175541043266	-1.95008575445650
H	11.69239472765253	2.82762920041983	-1.90631955773570
H	12.82969923508031	3.71792806066772	0.27800678657303
H	13.96931921147872	2.39634966655906	0.06069864579381
H	13.12625266416883	2.58150349799013	1.59665480774699
H	9.31605845370622	-0.16159084037212	-1.39377142218342
H	9.24846687526630	-1.60687195087822	-0.38737105138740
H	10.77947188355978	-0.76112286135489	-0.60559570139458
H	7.37519624823269	0.64381418853365	-0.00398074113514
H	7.53762367216330	0.92058866990327	1.73073304738728
H	7.44933783774859	-0.72426587066979	1.09736328429889
H	8.29949257508455	2.66981662990676	3.13618200625625
H	9.35269406732438	2.34747078263845	4.50662881601250
H	8.89902462957646	1.03342774489350	3.42273069331787
H	11.77253914917859	4.10130774139940	2.41603902823857
H	10.96900469546103	3.98472781888101	3.97824044416253
H	10.07741657340439	4.56225710097994	2.57466216168352
H	9.37543716189964	8.30926735458146	-0.04284899384737
H	7.64444557066068	8.60315144272345	0.05616162800390
H	8.39039856192571	8.24107282386902	-1.50022860661199
H	9.15068454731477	6.56266104998029	1.76995518607634
H	8.03146579366394	5.21520393459812	1.59436537931603
H	7.41213864654043	6.85212979594701	1.84213242713702
H	11.09006479130048	6.71792033429530	-3.26111045564438
H	12.44936996646059	7.18243247480071	-2.24118604057726
H	10.86081528892442	7.88692184084727	-1.96240721535143
H	11.38344008129114	5.41978393420274	0.86426657259765
H	11.20489035393061	7.12571456897153	0.48495263270344
H	12.70797330216992	6.29926558949281	0.09490679033293
H	8.92791520703648	7.22680908693213	-3.76578820208137
H	7.34516400349824	7.09307670594794	-2.99718327575652
H	7.61920012944047	6.37759780272570	-4.58214250643266
H	10.30479055179384	4.91429882675990	-4.14320066751392

H	8.84450050461474	4.23992599799747	-4.85708067208989
H	9.60083498929848	3.43752941133172	-3.47483537491841
H	3.50323533438607	7.42886905621856	-1.29214221331581
H	5.22961405208556	7.72974824352235	-1.43321932755208
H	4.61338312123565	6.69888538314843	-0.13047529262664
H	4.61665433186348	5.35369926378284	-4.25348556749960
H	5.00455729911024	7.01689231772370	-3.82407390612351
H	3.34294715739731	6.44827109810579	-3.71910362816806
H	1.65713081641553	6.57388461851827	-0.49640376837426
H	2.04887679434487	5.37928047067827	0.73155621823176
H	0.39741841806311	5.50178893944737	0.10230972698342
H	1.33299710571342	5.90118760201697	-2.92112876745994
H	-0.04348453756748	4.94481202605558	-2.38795577504947
H	1.21966596809900	4.19865957890836	-3.36711051669614
H	5.91563174399793	3.42129096306579	-3.35436482565909
H	5.36651759516401	1.90208558816964	-4.05652801125975
H	5.67390706511364	2.00821241759954	-2.32689026903105
H	1.88472997457766	1.95863005475528	-2.71069828217145
H	3.23384980803465	1.12037313387800	-1.94025854345753
H	3.04840132607422	1.06490650255416	-3.68865191741946
H	2.78979422070436	-0.72157674519670	2.48716415303255
H	3.85485452656297	-0.06951337509942	3.73290406919057
H	4.39077383622061	-1.41215123611827	2.72265313349590
H	3.39719996752988	-0.36068766631776	-0.03547175953459
H	4.98977402907620	-1.04260407081139	0.27683831204983
H	4.84258746203615	0.56603511467413	-0.43780172922721
H	1.27029810935939	0.90615686618954	3.31136133715379
H	-0.03300183993969	1.99806683554940	2.85429420267773
H	1.33566759573692	2.60093145641368	3.78658419309764
H	1.71855375716203	1.49243751987556	-0.39444198976183
H	0.14776519522345	1.50425588210174	0.41015099230656
H	1.34523686669678	0.28510494023218	0.82845725697696
H	6.00348420733398	1.86928628111570	3.76266838305284
H	5.83623800676437	3.29155945466129	4.79035667735769
H	6.14271102148196	3.49099613935101	3.06956178189806
H	4.06917482943636	5.03738291583094	3.04255711736536
H	3.77809768657716	4.69371214955173	4.75373603757591
H	2.50216951956787	4.42928526724835	3.56257509039481

S5.2.5 – Coordinates for **3’**

127

P	9.04307587808578	3.10450411463673	-0.65485618833792
Si	10.26104354928079	2.05714773206661	0.90942447781683
Si	9.38726480487150	5.21059590103786	-1.40218730457487
C	12.02970602233779	1.77847420600087	0.23435481557055
C	9.45731514167622	0.32052476272048	1.01085493815860
C	10.37200144601749	2.69652507416262	2.70271631395083

C	11.22021088878467	5.70338837084890	-1.49292151291094
C	8.62659763791202	4.96460412537181	-3.13808244733161
C	8.32227110846516	6.50650111831599	-0.48103551089694
Cd	6.72701126253277	3.43472025019939	-0.04384597299345
P	4.41885577295123	3.76380873525613	0.56507767696218
Si	3.50120305617376	4.40006243835856	-1.37773867412912
Si	3.53197917836444	2.08411680527310	1.76586122313873
C	1.64754560746231	4.68853386983936	-1.07368580524116
C	4.51955768775991	5.96186667576212	-1.79936264935939
C	3.67299694882840	3.27103675252186	-2.90283941476533
C	1.63471658590268	2.01545333118448	1.58762345768865
C	3.91567953285441	2.63166748901709	3.54856993863722
C	4.39247172551926	0.41855641862727	1.47595679362816
H	3.27772904093113	3.89917404343535	-3.71359130667333
C	2.83684652978998	2.00065477127028	-2.86336456234015
C	5.11977194714035	2.94712328753973	-3.24492088973057
C	4.20030778364671	6.56526770139222	-3.16439556459453
H	5.54812340089429	5.57068226225975	-1.85707084567360
C	4.50515031771207	7.04097288186783	-0.72354961819598
C	0.88648101004718	5.13914829689711	-2.31829104402840
C	1.33643810459704	5.59982576769399	0.10903532306023
H	1.28538704604097	3.68452862377501	-0.81774130052258
C	4.45303154175380	-0.04752987327848	0.02909402796721
C	3.85968069098647	-0.68357100317672	2.38827053510917
H	5.42200143552653	0.62945320460633	1.78685102630771
C	1.15196126070404	1.18811792722656	0.40182232198413
H	1.32931213829688	3.05587149763472	1.42450217226545
C	0.94454352412539	1.52808795034453	2.86185340513877
C	5.40743871892683	2.64983880087488	3.85397626361555
H	3.44399514671529	1.87566007744614	4.19066045569550
C	3.30261547213121	3.98998531262259	3.87597269749405
C	9.86007779910575	-0.63525595244310	-0.10708444444202
C	7.93860878296982	0.33434126258432	1.11668897047296
H	9.85955051839804	-0.07722461660399	1.95507407466653
C	10.81862447903707	4.14285958245681	2.85731660420140
H	11.15458089168144	2.04998889415133	3.13040890281939
C	9.09882195201044	2.47462460481552	3.50905230062453
C	12.09439378327225	1.68574037955416	-1.28644485252313
H	12.25976921700150	0.78139631863673	0.63948586247230
C	13.11475438355599	2.71896206653004	0.74364792965324
C	8.59195784786490	7.94628031640930	-0.91890296618852
C	8.33379802884998	6.43222033553251	1.03978275968041
H	7.30182502946731	6.25827669651319	-0.81221565080695
H	11.74612004597550	4.77747538205908	-1.75538329920423
C	11.75739401980631	6.18515563926483	-0.15029613729776
C	11.52738824745829	6.73217454992206	-2.57993114627172
C	9.49692563817686	4.07090072494276	-4.01697717759101
C	8.17317932787907	6.20912488404090	-3.89250530947160

H	7.72214749107352	4.38347983746918	-2.90896160444651
H	11.34682419078631	1.01056768088053	-1.70238818596023
H	13.08297478617853	1.34123673654057	-1.60891169204562
H	11.92307965382184	2.66412094128242	-1.74106720223111
H	12.95339803731167	3.74379842245596	0.40264449114325
H	14.09255552283551	2.40539501144275	0.36225156018512
H	13.17909089129193	2.73251014820843	1.83314887316355
H	9.50104451765814	-0.27319280928525	-1.07447373740674
H	9.41320597734627	-1.62175539948818	0.05732834470888
H	10.93975631731126	-0.77341323239173	-0.17992938351210
H	7.49417740189121	0.60523931137320	0.15424442609017
H	7.56920089530881	1.03375923616400	1.86786175761825
H	7.55284532044998	-0.65839147184789	1.37331919082067
H	8.25699187946235	3.02443733208922	3.07771716714347
H	9.22551670311866	2.84105181650657	4.53320730319672
H	8.81557510402404	1.42315307370438	3.57056909693600
H	11.78995005503456	4.33667535326301	2.40454634729764
H	10.89206644973009	4.40801513062980	3.91756593766601
H	10.09467131367017	4.82107187706185	2.40284676347207
H	9.56563039043704	8.28966745942114	-0.56142560441108
H	7.84034250464714	8.61967247803915	-0.49319711221823
H	8.57502570622895	8.07406246995321	-2.00107619688862
H	9.29882771588716	6.74732387728965	1.44366704764444
H	8.14388871915073	5.42165439711302	1.40967913667381
H	7.56876487459690	7.09082919638965	1.46344318137500
H	11.24727433782972	6.38593876445594	-3.57515912557603
H	12.60033051920744	6.95217041475130	-2.59911922304341
H	11.00930116973401	7.67662530248955	-2.39944411691529
H	11.52306565195899	5.49583925553946	0.65958805166271
H	11.33427461442692	7.15671705400617	0.11634537669590
H	12.84519327237603	6.30701097081758	-0.18537198927030
H	9.00532612779750	6.87875584701817	-4.12364464863002
H	7.42948025876727	6.78129019564759	-3.33417390594306
H	7.71353976319125	5.92748309546916	-4.84631981023998
H	10.41554670999119	4.57732140645183	-4.32383675770825
H	8.96332011937718	3.79013952677528	-4.93102402560353
H	9.77667231114247	3.15176422113058	-3.49718105437990
H	3.52787512447509	7.52658409786675	-0.66996064346028
H	5.24003586753972	7.82183800584727	-0.94820610691990
H	4.72675917583916	6.63554203162197	0.26566684874211
H	4.28100213973599	5.83980801474756	-3.97620303837427
H	4.88864798296288	7.38648045585561	-3.39043387814206
H	3.18899077772841	6.97851392977520	-3.18858663365979
H	1.55681422734911	6.64318382782853	-0.12854372477651
H	1.91930241151386	5.33880521918334	0.99507506289153
H	0.27422102167606	5.54631916784259	0.37072481308281
H	1.17238984035187	6.15083751375885	-2.61672676232231
H	-0.19137646565967	5.15464512154016	-2.12452310331190

H	1.05522448043299	4.48266334465305	-3.17478602045589
H	5.72923448500261	3.84469305835702	-3.36936349838250
H	5.18783484547935	2.37646864089247	-4.17696041652130
H	5.58383194528083	2.33258482732112	-2.46601623186421
H	1.77602600291645	2.20618348981823	-2.70429400393175
H	3.17267015251287	1.33271763443775	-2.06790881882314
H	2.92619765142081	1.45021832835528	-3.80606117857777
H	2.84223139851412	-0.97356821647800	2.11266923389397
H	3.84488638769439	-0.38390420349396	3.43895552759718
H	4.48076709941733	-1.58246810156198	2.31174021440292
H	3.46546022497463	-0.32043704627507	-0.34955842559546
H	5.09432609941328	-0.92961408528137	-0.06948640673115
H	4.85243564943994	0.73118410643892	-0.62467671553814
H	1.21541216906074	0.49518473838610	3.09549181542035
H	-0.14285148043452	1.55392891430459	2.73284365912653
H	1.18517072170869	2.13702471573987	3.73377128622250
H	1.62922308853070	1.47358515610134	-0.53457046964721
H	0.06974769553471	1.29454447807884	0.27092393042386
H	1.35174193594973	0.12483975095099	0.55836579555481
H	5.87869240241096	1.67341068929436	3.72148048217748
H	5.59360660019792	2.96535621986119	4.88598605839545
H	5.92301615607394	3.36459983968227	3.20475879899821
H	3.77741107799155	4.77398563777036	3.28045335225541
H	3.44748562674848	4.23733333362834	4.93295633489336
H	2.23017926690037	4.03195166433124	3.67105051321009

S5.2.6 – Coordinates for **4’**
127

P	9.02349380016152	3.08588542717942	-0.61740682853065
Si	10.24852392508185	2.08787099420128	0.98254724255788
Si	9.37315131337848	5.18013511743122	-1.41765396521319
C	12.01300621754542	1.80293465477404	0.29820086857141
C	9.46313341491190	0.34803682136549	1.13521808026720
C	10.36582280098768	2.78023773520621	2.75462028247492
C	11.20999954494772	5.65442336792956	-1.53288167426556
C	8.59844661522773	4.88774508710835	-3.13827483628822
C	8.32861746088559	6.50762733145917	-0.52239851342839
Hg	6.72114989764775	3.42353792141112	-0.02598285028349
P	4.42781476678542	3.75986922579710	0.56312693784439
Si	3.50570145641659	4.44049259379055	-1.37052402715799
Si	3.54717026450633	2.03917975718029	1.72107218011949
C	1.65729755019315	4.72306981354366	-1.02895722247847
C	4.52287363973073	6.00820616412870	-1.76267682798531
C	3.65254697544762	3.35095247898262	-2.92530471451090
C	1.65101461411800	1.97382632448990	1.53246861765317
C	3.91724066973036	2.54755910267210	3.51761220341276
C	4.41704016687453	0.38810371697111	1.39084973222959

H	3.22477169368884	3.99488583548918	-3.70693706234171
C	2.83417374601025	2.06912214090448	-2.89599936172194
C	5.09270765232054	3.05627989056356	-3.31842135872392
C	4.17279566981342	6.65419545651485	-3.10052953846689
H	5.54495806232594	5.60945787537272	-1.85678056829670
C	4.54346847220326	7.05442330142375	-0.65517112913806
C	0.87504160400142	5.19702397738861	-2.25175702016356
C	1.36750658434190	5.61438270282108	0.17453986837765
H	1.29750816124705	3.71534005652020	-0.78505598681015
C	4.49046593706480	-0.03995003532418	-0.06722837923654
C	3.88602066724501	-0.73957903693701	2.27257954924710
H	5.44265645494552	0.59739354724980	1.71371501649360
C	1.17530231433664	1.17374684408852	0.32552880273960
H	1.34273829424862	3.01707311059896	1.39461397436475
C	0.95755372031009	1.45294972743728	2.79178183030767
C	5.40607301665256	2.55895888728075	3.83573354708533
H	3.44053316311793	1.77691703891763	4.13832742779569
C	3.30061315691662	3.89779497246743	3.87119958204423
C	9.87298471817520	-0.63807307215894	0.04644370533438
C	7.94478250930563	0.35124204358291	1.24117983323468
H	9.87036837855995	-0.01613704598843	2.09088280689606
C	10.81359797285561	4.23014231871007	2.86591170032921
H	11.14961243490263	2.14557675325321	3.19780440282155
C	9.09487090082072	2.58265771534642	3.57124793374302
C	12.06916193068659	1.66782034539264	-1.21982297700584
H	12.24581630710426	0.81692512100913	0.72802173261656
C	13.10214187890726	2.75451807157643	0.77686635021746
C	8.60670351318631	7.93250873378480	-1.00158042660244
C	8.35144826010564	6.47231362336605	0.99954572071689
H	7.30468829330534	6.25719046179561	-0.83665450997874
H	11.73168658026466	4.71984666505761	-1.77130380963836
C	11.75883626794101	6.17592948346399	-0.20992505855350
C	11.51298192680987	6.64935426659901	-2.65249833432056
C	9.45747046457813	3.97007389629591	-4.00311844484508
C	8.13941962565196	6.11319638108302	-3.91991974210239
H	7.69598942437079	4.31570325882502	-2.88238848146986
H	11.32025249943310	0.98037118171783	-1.61237988213140
H	13.05629013572676	1.31481677345310	-1.53746264456074
H	11.89600704788908	2.63260182060513	-1.70182317590353
H	12.94230247079652	3.77033206148780	0.41006191332162
H	14.07707855517477	2.42777483811536	0.39922653433423
H	13.17180636367507	2.79638474938400	1.86528105889785
H	9.50625675808034	-0.31096096228002	-0.93046376410707
H	9.43612980133078	-1.62296706366106	0.24393484447559
H	10.95358418824019	-0.76866901398893	-0.02676463388009
H	7.49836919818307	0.58590319541776	0.27059084950082
H	7.56921438847228	1.07494265056300	1.96546940746431
H	7.56932676249188	-0.63591971436978	1.53257892374434

H	8.25361581520286	3.12349632084957	3.12835165320247
H	9.22773255215342	2.97485246849407	4.58500099103342
H	8.80885898153811	1.53400925472112	3.66111738994989
H	11.78270038897458	4.41162888733611	2.40333076186926
H	10.89252390735541	4.52443809193236	3.91802547891181
H	10.08703711761937	4.89527673084061	2.39696593044559
H	9.58579017245808	8.27930519521132	-0.66270908341700
H	7.86372883336382	8.62220717477594	-0.58681397246473
H	8.57943116295096	8.03282199616723	-2.08659548324140
H	9.32508152547180	6.77841606581751	1.38935105104071
H	8.13855870203521	5.47670206503184	1.39434383280471
H	7.60320935517838	7.15858160150672	1.40946376693146
H	11.22903751530750	6.27297483219908	-3.63561459872612
H	12.58582629449648	6.86857063379751	-2.68236890552353
H	10.99533866867458	7.59869046861630	-2.49868332820012
H	11.53078317341940	5.51257725687244	0.62299194043068
H	11.33966239240172	7.15586113674994	0.03039498402179
H	12.84649614914819	6.29473653131879	-0.25820438892562
H	8.97030530580050	6.77462492908451	-4.17783117203769
H	7.40313541685660	6.70158003958117	-3.36855822599402
H	7.66869729092884	5.80712129128096	-4.86075288756538
H	10.37214100450226	4.46734368244458	-4.33557004343959
H	8.91160677391504	3.66482555827840	-4.90192636287189
H	9.74367814349630	3.06487069397121	-3.46267716236620
H	3.57202222040225	7.54565170412449	-0.56141363244525
H	5.27922684550656	7.83577688880961	-0.87517286705141
H	4.78725714152282	6.61859273769411	0.31559385953927
H	4.22336146843930	5.95164754142264	-3.93461958334359
H	4.86433330412718	7.47416365661841	-3.32160213805750
H	3.16605221677356	7.07901808980317	-3.08538394359003
H	1.59009351009235	6.66068511179176	-0.04745342328046
H	1.96084442585162	5.33533985400441	1.04803112580259
H	0.30873927187119	5.56144668526596	0.45003421384118
H	1.15879072610374	6.21257901664250	-2.53877607265176
H	-0.19883661232897	5.21253659068197	-2.03714124053252
H	1.02533179383629	4.55429565101886	-3.12185458148281
H	5.67948419708372	3.96634742419099	-3.45647564955561
H	5.13617281620091	2.49512337042529	-4.25773511978272
H	5.59622180888678	2.44702529542563	-2.56086140912429
H	1.77459976591115	2.25636189454392	-2.70848049066941
H	3.19751080635813	1.38765921818461	-2.12445187712568
H	2.90893217378272	1.54229553912519	-3.85331081414598
H	2.87151884479105	-1.02884223653225	1.98548148860875
H	3.86540863653682	-0.46635484447722	3.33038671075003
H	4.51318927739948	-1.63232262359870	2.17635095472634
H	3.50744839954938	-0.30906401384805	-0.46030689999643
H	5.13744854926462	-0.91599689199213	-0.18170106658747
H	4.89210624879817	0.75634167449919	-0.69803111805908

H	1.23171556171552	0.41572553366503	3.00105329446647
H	-0.12926316545339	1.47757896019070	2.65819204510343
H	1.19169969193262	2.04136409397248	3.67934154176069
H	1.65594291743233	1.48166855518106	-0.60158969647488
H	0.09336954796506	1.28062707571761	0.19277522329634
H	1.37718991824274	0.10760982276808	0.45825733230649
H	5.87569982052318	1.58352728347411	3.69083079084252
H	5.58331802240032	2.85644500593029	4.87461868221733
H	5.92874137021528	3.28311049144564	3.20314757558976
H	3.78157502682825	4.69621506792064	3.30033757613295
H	3.43502765467916	4.11899577652863	4.93528021277459
H	2.23022613110875	3.94527262666079	3.65691111923606

S5.2.7 – Coordinates for “bent” $Cd[P(SiMe_3)_2]_2$
55

Cd	-0.12160650129610	0.08761009198037	-0.00977788705445
P	-0.08031343854502	-0.50271677095727	2.34134939094914
P	-0.25267806587185	0.73189901923699	-2.34225680119604
Si	-2.08576268839317	-0.48444817638880	-2.80817103389143
Si	2.15627959992823	-0.44044375257754	2.57891500836096
Si	-0.75471082244005	1.52348995778815	3.04831459772366
Si	1.45242301526878	-0.53289176565471	-3.08797439514563
C	2.74529436091438	-2.20560158397808	2.34504377577595
C	-2.57597196651116	-0.13754875309538	-4.58423495577225
C	-1.92092977367305	-2.34097623969702	-2.57337576517205
C	3.05467512357454	0.63750101464874	1.32413493711365
C	-3.44489369956906	0.13427902214568	-1.66765849264507
C	-0.90323242082061	1.47590102354974	4.91673857898483
C	2.61636064829440	0.14239727303470	4.30142612520633
C	1.81746039427155	-2.06251179681160	-2.05323491223465
C	2.96670767472478	0.57248050016531	-3.04709627116709
C	-2.45978600576962	1.80722881879573	2.31145367328067
C	1.13602162555466	-1.06230941185189	-4.85982697610077
C	0.34259285999579	2.96961665405081	2.56143509751872
H	-3.52457632554087	1.22310218445890	-1.70911027472933
H	-3.26249727679530	-0.15042768803006	-0.62737403147979
H	-4.41148448935716	-0.28740407079264	-1.95877800950608
H	-1.15227208922862	-2.76691008267212	-3.22148830325541
H	-2.86910809961938	-2.83123748546593	-2.81469134204731
H	-1.66796407726697	-2.59242278112105	-1.54019319293185
H	-1.80079979024454	-0.45988887560323	-5.28232310284689
H	-2.73660834609782	0.93234643442553	-4.73466037117601
H	-3.50170876424628	-0.66257212402802	-4.83795708804436
H	2.02535354385634	-1.55219969180669	-5.26737573674537
H	0.90699400009567	-0.19868704852714	-5.48820798358411
H	0.30374198153482	-1.76529623294173	-4.93354413575213
H	3.85081100008518	0.03372413839864	-3.40046325143944

H	3.17170752753116	0.93069766170120	-2.03522617164618
H	2.82062684504701	1.44758279429607	-3.68424499694205
H	2.93697912719555	0.24579146846555	0.30936189484229
H	4.12768689392697	0.65434370839461	1.54042143031120
H	2.69337021155719	1.66752381977341	1.33266526975337
H	0.42339649295210	3.06545552839739	1.47541073734049
H	1.35174315419902	2.87301513974832	2.96681417610269
H	-0.08302276732391	3.90155400500199	2.94655848496038
H	-2.41460372386000	1.96330874332007	1.22973643466888
H	-2.92483076697233	2.69399361382106	2.75217039674952
H	-3.11367329943852	0.95260872659868	2.50027077577572
H	-1.57001944867171	0.66847746901450	5.22753341825996
H	-1.30888588677130	2.41957357058899	5.29337886340970
H	0.06659403249742	1.31023368579936	5.38978857215171
H	2.63061301371324	-2.63933082791060	-2.50510586539021
H	0.94631987032358	-2.71468031962104	-1.96920562287624
H	2.13496989515999	-1.79154831183542	-1.04207024835592
H	2.47652932263505	-2.58202285063617	1.35468876611554
H	2.29015670151790	-2.86714788157440	3.08534976383756
H	3.83247658463069	-2.27037790377576	2.44755201526253
H	3.69275814900799	0.02460269147566	4.45817104063875
H	2.09636194485064	-0.43935872760648	5.06560618392001
H	2.37061493947977	1.19572239588456	4.45127781011390

S5.2.8 – Coordinates for “linear” $Cd[P(SiMe_3)_2]_2$
55

P	8.76219230072295	2.90347759652652	-1.01102563825589
Si	9.79013155767071	2.48716556593112	0.94615766580309
Si	9.35609720211546	5.01920092481832	-1.50377129451143
C	11.55069151251431	1.95698901131070	0.58108066861792
C	8.87295832862027	1.05195235258675	1.74223982057621
C	9.82565982849067	3.90589758547670	2.17719089838676
C	11.21935004250695	5.21410542015788	-1.40469159353126
C	8.79961558961293	5.29590706976738	-3.27385422284173
C	8.55403272499129	6.34466550688453	-0.43607284700873
Cd	6.63422306342861	3.46436165385431	-0.00190751947382
P	4.50774005423866	4.02798484128749	1.00883210992391
Si	3.47875759591212	4.44220044349426	-0.94837330148467
Si	3.91156152715752	1.91376230359739	1.50535580453666
C	1.71897278885735	4.97512199037252	-0.58347297736639
C	4.39702167434866	5.87489302613499	-1.74774320267483
C	3.44058685629497	3.02118414507120	-2.17670906025809
C	2.04807524374578	1.72038901108030	1.40778969017621
C	4.46875714920762	1.63923591630044	3.27555919686795
C	4.71197659801977	0.58588255742921	0.43941860617873
H	2.92951672546516	3.33636638300698	-3.09181949903396
H	2.90664319560628	2.15513546357245	-1.77994873439157

H	4.44781305698004	2.69773669915962	-2.45226433042882
H	3.84512025368810	6.24720561723998	-2.61582903402527
H	5.38985326934645	5.57421643511628	-2.09538755394982
H	4.52314195998050	6.70214842908658	-1.04538617530959
H	1.19960230996144	5.24736206253473	-1.50710283966141
H	1.71021607076064	5.84077092098551	0.08261063455061
H	1.15433362918873	4.17544145794818	-0.09987249314437
H	4.50883063244883	0.73160332545785	-0.62313127495792
H	4.33050917620808	-0.40032202236773	0.72320623969508
H	5.79744628312098	0.56350117657561	0.57493916017236
H	1.68844759732123	1.77730565946804	0.37816281122253
H	1.54219265601115	2.49539151199087	1.98776198538046
H	1.75315405218558	0.74695684410275	1.81092152430605
H	5.55052709025318	1.76531612261363	3.37054845613919
H	4.21767586668202	0.62846089145971	3.61012289343888
H	3.99117888897996	2.35362492141686	3.94965862921242
H	8.74836397283910	0.22580261030822	1.03830908171157
H	7.87948729232961	1.35090352419683	2.08954340960430
H	9.42455599486502	0.67872511891356	2.61012551337940
H	10.35866415225974	4.77347731328023	1.78252638612197
H	10.33646420924396	3.58962187373047	3.09206889562527
H	8.81773841637423	4.22734257245628	2.45255998090605
H	11.56062357665533	1.09295880963816	-0.08708611983794
H	12.06977558329354	1.68302172413204	1.50436108627623
H	12.11485928638155	2.75835653523775	0.09973198079663
H	8.93514121366496	7.33156247750599	-0.71793365537107
H	8.75665531460912	6.19719486087681	0.62633302290971
H	7.46863319069243	6.36670844514885	-0.57217310796153
H	11.72642311705699	4.44064708947623	-1.98567862869891
H	11.57821191239846	5.15556320722257	-0.37488079416685
H	11.51366320235902	6.18858043346678	-1.80574299475834
H	9.27799548125573	4.58277581771977	-3.94871445278049
H	9.05016585807544	6.30731572413446	-3.60690205132412
H	7.71797387300059	5.16930304110492	-3.36963075530732

S6 – References

(1) Rigaku Oxford Diffraction, (2018), CrysAlisPro Software system, version 1.171.40.45a, Rigaku Corporation, Oxford, UK.

(2) Dolomanov, O. V.; Bourhis, L. J.; Gildea, R. J.; Howard, J. A. K.; Puschmann, H. OLEX2: A Complete Structure Solution, Refinement and Analysis Program. *J. Appl. Crystallogr.* **2009**, *42* (2), 339–341. https://doi.org/10.1107/S0021889808042726.

(3) Sheldrick, G. M. SHELXT – Integrated Space-Group and Crystal-Structure Determination. *Acta Cryst. A* **2015**, *71* (1), 3–8. https://doi.org/10.1107/S2053273314026370.

(4) Sheldrick, G. M. Crystal Structure Refinement with SHELXL. *Acta Crystallogr. C Struct. Chem.* **2015**, *71* (Pt 1), 3–8. https://doi.org/10.1107/S2053229614024218.

(5) *CheckCIF*. https://checkcif.iucr.org/ (accessed 2024-07-15).
